# Molten‐Salt‐Mediated Synthesis of Atomic Manganese/Cobalt Catalysts on Bioceramic Microparticles for Catalytic Anti‐Osteoarthritis Treatments

**DOI:** 10.1002/advs.202505500

**Published:** 2025-07-21

**Authors:** Ronghui Deng, Zining Zhang, Aijun Wu, Chaoqin Shu, Shitang Song, Fuzhen Yuan, Zijie Xu, Meng Yang, Jing Ye, Yifan Song, Yufang Zhu, Jia‐Kuo Yu

**Affiliations:** ^1^ Sports Medicine Department Beijing Key Laboratory of Sports Injuries Peking University Third Hospital Beijing 100191 P. R. China; ^2^ Peking University Institute of Sports Medicine Beijing 100191 P. R. China; ^3^ Orthopaedic and Sports Medicine Center Beijing Tsinghua Changgung Hospital Tsinghua University Beijing 102218 P. R. China; ^4^ State Key Laboratory of High Performance Ceramics and Superfine Microstructure Shanghai Institute of Ceramics Chinese Academy of Sciences Shanghai 200050 P. R. China; ^5^ Center of Materials Science and Optoelectronics Engineering University of Chinese Academy of Sciences Beijing 100049 P. R. China

**Keywords:** antioxidant, bioceramic, microparticles, molten‐salt method, osteoarthritis

## Abstract

Osteoarthritis (OA) is a chronic progressive joint disease characterized by cartilage degeneration and local inflammation, and its progression is closely related to the excessive production of reactive oxygen species (ROS). Despite progress made with small molecule antioxidants and nanozymes, effective antioxidant therapy for the long‐term elimination of these ROS remains challenging, largely due to the rapid clearance of antioxidants from the joints via synovial vessels and lymphatics. Herein, a molten‐salt method is developed to facilitate the atomic dispersion of Mn or Co ions homogeneously on the surface of akermanite microparticles (AKT‐MPs). The micrometer‐scale Mn‐ or Co‐AKT‐MPs with multi‐mimetic enzyme effects are demonstrated to obliterate multiple ROS, thereby protecting the inherent homeostasis between chondrocyte anabolism and catabolism, while suppressing the conversion of macrophages to a pro‐inflammatory phenotype. In addition, the microparticles exhibited chondroprotection of ROS‐challenged cartilage explants in vitro by limiting the loss of cartilage extracellular matrix (ECM) and the release of degradative enzymes. Furthermore, Mn‐ or Co‐AKT‐MPs are injected intra‐articularly into monosodium iodoacetate (MIA)‐induced OA mice and effectively suppress synovial inflammation, painful symptoms, and progression of early cartilage destruction. Therefore, this microparticle‐based antioxidant therapy provides an insight and paradigm to control atomic catalysts integrated with microparticles for efficient catalytic anti‐OA treatments.

## Graphical abstract

Microparticle‐based antioxidants capable of scavenging multiple reactive oxygen species (ROS) have been developed. When retained within the articular cavity of osteoarthritic mice, Mn‐ or Co‐AKT‐MPs protect the inherent homeostasis between cartilage anabolism and catabolism, and inhibit synovial inflammation.

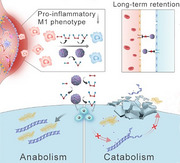

## 1 Introduction

Osteoarthritis (OA) is the most prevalent chronic joint disease, characterized by progressive destruction of articular cartilage and accompanied by painful symptoms and loss of function that impairs quality of life.^[^ ^1^ ^]^ However, the current existing nonoperational therapies for OA are generally aimed at pain relief in order to preserve the residual joint function.^[^ ^2^ ^]^ There are no US Food and Drug Administration‐approved OA treatments or interventions with proven effectiveness in reversing the pathological changes in OA.^[^ ^3^ ^]^ Cartilage degeneration is the most prominent pathological feature of OA, along with pathological changes in other joint components such as subchondral bone, synovial fluid, synovium, and the local immune system.^[^ ^4^ ^]^ While the molecular mechanisms underlying the onset, development and progression of OA remain elusive,^[^ ^5^ ^]^ local inflammation plays a critical role throughout the course of the OA pathogenesis,^[^ ^6^ ^]^ leading to excessive production of multiple reactive oxygen species (ROS) such as peroxide (H_2_O_2_), hydroxylated radical (·OH), and superoxide radical (O_2_·^−^).^[^ ^7^ ^]^

Under healthy conditions, ROS are generated at low levels and their harmful effects are normally countered by the natural antioxidant defense systems such as superoxide dismutase (SOD), catalase (CAT), and glutathione peroxidase (GPx).^[^ ^7^, ^8^ ^]^ However, under the pathological condition of OA, the balance between ROS and antioxidants is disturbed due to downregulation of endogenous antioxidants and excessive accumulation of ROS.^[^ ^9^ ^]^ Oxidative stress in the osteoarthritic microenvironment induced by high levels of ROS results in protein carbonylation, hyperoxidation, and DNA damage, thereby compromising chondrocyte function and even leading to senescence and apoptosis.^[^ ^10^ ^]^ It also affects various cell signaling pathways to promote the overexpression of many pro‐inflammatory factors and matrix‐degrading enzymes (for example, PGE2 and MMP13), which in turn impede cellular anabolism and deteriorate the degeneration of cartilage extracellular matrix (ECM).^[^ ^11^ ^]^ In addition, the overproduced ROS can also trigger the activation of the local immune system within the joint cavity, such as the programming of macrophages to a pro‐inflammatory M1‐like phenotype, secreting many of the inflammatory mediators (for example, TNF‐α, IL‐1β, and IL‐6), which further exacerbates cartilage damage and function loss.^[^ ^12^ ^]^ Therefore, scavenging ROS could be a potential treatment strategy for limiting OA progression.

Currently, treatments such as antioxidant enzymes, various small molecule antioxidants, and antioxidant nanomaterials are being explored to target the oxidative stress existing in the pathogenesis of OA.^[^ ^13^ ^]^ Antioxidant enzymes have had variable success in animal or clinical testing to treat OA. SOD and CAT, for example, have been shown to provide only modest protection against oxidative damage because of their intrinsic properties, namely rapid inactivation of free proteins, inability to scavenge multiple ROS, and antigenicity.^[^ ^8^, ^14^ ^]^ Although antioxidant molecules, such as vitamin E, glutathione, curcumin, and so on, are also available, their low bioavailability, short biological half‐life, and instability under physiological conditions fail to dampen a complex joint inflammatory response.^[^ ^15^ ^]^ Alternatively, nanozymes with enzyme‐like properties have emerged as potential candidates, successfully mimicking the antioxidant enzymes of SOD, CAT and GPx and scavenging O_2_·^−^, H_2_O_2_, and ·OH in inflammation‐related pathological abnormalities.^[^ ^16^ ^]^ However, even with local intra‐articular injections, both nanozymes and small molecular antioxidants exhibit rapid excretion from the joint via synovial vessels and synovial lymphatics within a few hours.^[^ ^15^, ^17^ ^]^ Consequently, administration of high doses or frequent injections is required to achieve therapeutic benefit.^[^ ^18^ ^]^ Thus, there is an unmet need to develop new antioxidant medicines capable of scavenging multiple ROS within the OA microenvironment, as well as featuring a prolonged mechanism of action to enable less frequent injections.^[^ ^19^ ^]^

Microparticles composed of diverse polymers have been extensively investigated as drug depots for joint delivery owing to their large size (1–1000 µm) and long‐term drug retention.^[^ ^20^ ^]^ In the United States, a PLGA microparticle loaded with triamcinolone acetonide, termed Zilretta, has received clinical approval for intra‐articular administration in OA.^[^ ^21^ ^]^ However, an antioxidant microparticle for prolonged scavenging of ROS within the joint has not yet been developed. Although cerium oxide, manganese dioxide, and cobalt‐based nanoparticles have demonstrated multi‐antioxidant ability against ROS, their activities are highly dependent on their sizes.^[^ ^22^ ^]^ Hence, it remains challenging to explore an ideal microparticle‐based antioxidant that not only exhibits reduced joint clearance but also robustly scavenges multiple ROS with high efficacy.

**Scheme 1:**
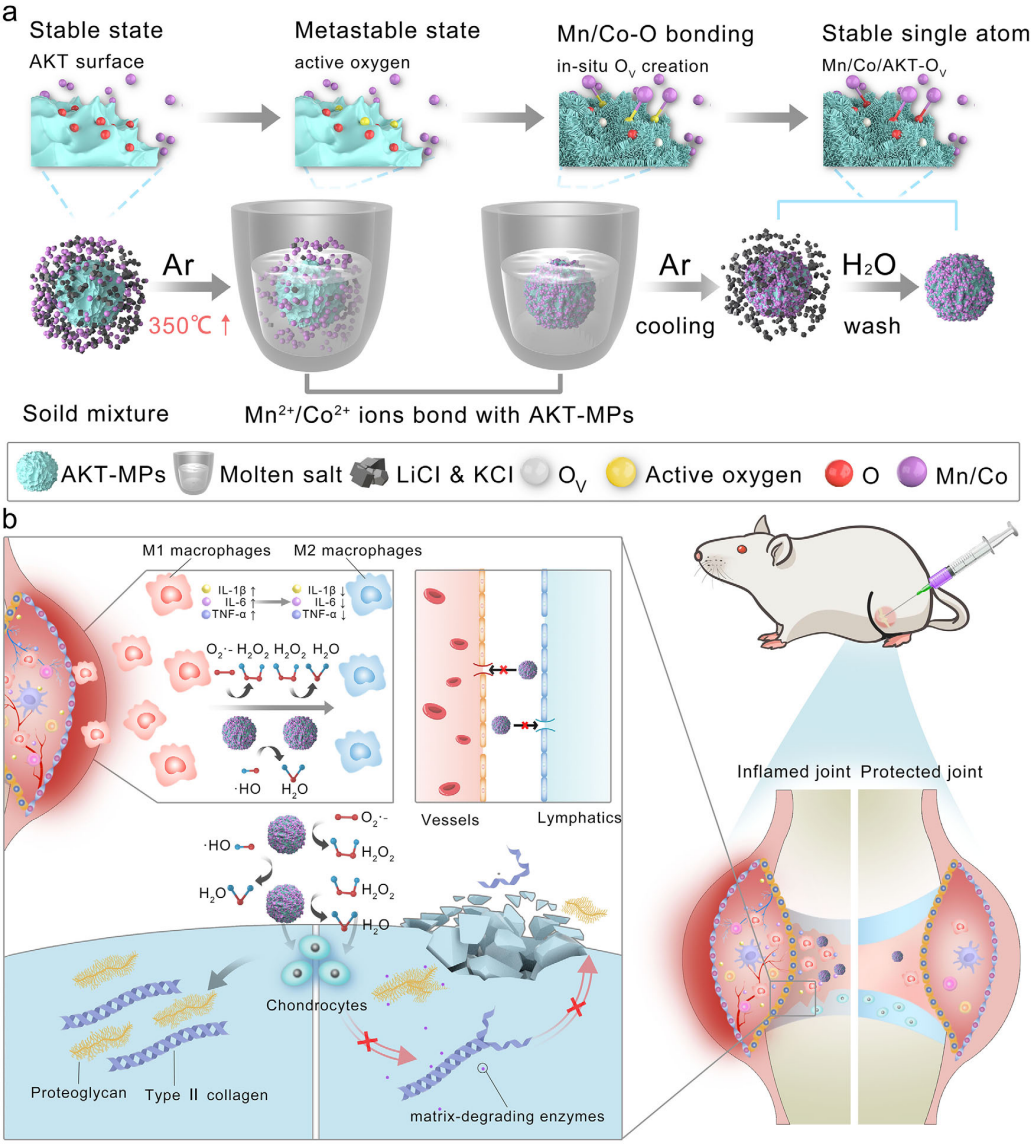
Schematic illustration of the synthetic process of Mn‐AKT‐MPs and Co‐AKT‐MPs and mechanisms of their activity in the treatment of OA. a) Atomically distributed Mn or Co catalysts on the surface of AKT microparticles are synthesized by a controlled molten‐salt method. b) After intra‐articular injection, Mn‐AKT‐MPs or Co‐AKT‐MPs are expected to sustainably eliminate multiple excessive ROS in an inflamed microenvironment in the long term, thereby orchestrating the inherent homeostasis between chondrocyte anabolism and catabolism, while suppressing the conversion of macrophages to a pro‐inflammatory phenotype under ROS stimulation.

Recently, decreasing the particle size of catalysts to the atomic scale is an effective strategy to maximize the catalytic efficiency because of the maximal number of surface active sites for catalysts.^[^ ^23^ ^]^ A good example is the atomic Co decorated single‐atom catalyst, of which the multi‐antioxidant activity is superior to the existing antioxidant nanozymes.^[^ ^24^ ^]^ In this regard, the synthesis of atomic catalysts on microparticles is highly promising to achieve efficient microparticle‐based antioxidant systems. The molten salt method is widely used in the preparation of optoelectronic materials, battery components, and catalytic substrates.^[^ ^25^ ^]^ Molten salts serve as high‐temperature solvents characterised by solubility, stability, and recoverability.^[^ ^26^ ^]^ These attributes not only accelerate reaction kinetics but also promote the desolvation of precursors, facilitating the formation of bare ions.^[^ ^27^ ^]^ Nevertheless, the use of the molten salt approach in the synthesis of bioactive materials remains unexplored. Inspired by this, we report the synthesis of atomically distributed Mn or Co catalyst on the surface of microparticles via a controlled molten‐salt method (Scheme 1a).^[^ ^28^ ^]^ As a model microparticle, we chose Akermanite (AKT), a typical bioceramic with good biocompatibility that has been extensively studied in the bone and cartilage tissue engineering field,^[^ ^29^ ^]^ which we ground and prepared it into microparticles before modification with either atomic Mn or Co. When the melting point of the salts (the mixture of LiCl and KCl) is reached, the liquid environment formed allows an atomic dispersion of Mn^2+^ or Co^2+^ homogeneously on the AKT microparticles. The strong polarizing force generated by the molten salt enables the formation of strong Mn─O or Co─O bonds on the surface of the AKT microparticles, which effectively prevents the aggregation of the Mn or Co atoms and the damage to the AKT structure.^[^ ^30^ ^]^ The obtained monodisperse Mn‐AKT‐MPs and Co‐AKT‐MPs could effectively eliminate H_2_O_2_ and O_2_·^−^ by mimicking multiple enzymes of CAT and SOD, while efficiently reduce ·OH through oxidative‐reduction cycle. In in vitro biological experiments, Mn‐AKT‐MPs and Co‐AKT‐MPs substantially delayed the catabolic metabolism of chondrocytes and cartilage explants when subjected to ROS stimulation. In addition, these microparticles demonstrated a significant ability to mitigate the inflammatory polarization observed in ROS‐exposed macrophages. Ultimately, when tested in the monosodium iodoacetate (MIA)‐induced OA mice, both Mn‐AKT‐MPs and Co‐AKT‐MPs reduced the excessive ROS levels and inhibited the production of pro‐inflammatory cytokine, endowing a distinct cartilage protection to OA mice (Scheme 1b). Our results demonstrated that Mn‐AKT‐MPs and Co‐AKT‐MPs are promising microparticle‐based antioxidants for OA treatments.

## 2 Results and Discussion

### 2.1 Preparation and Characterizations of Mn‐AKT‐MPs and Co‐AKT‐MPs

**Figure 1:**
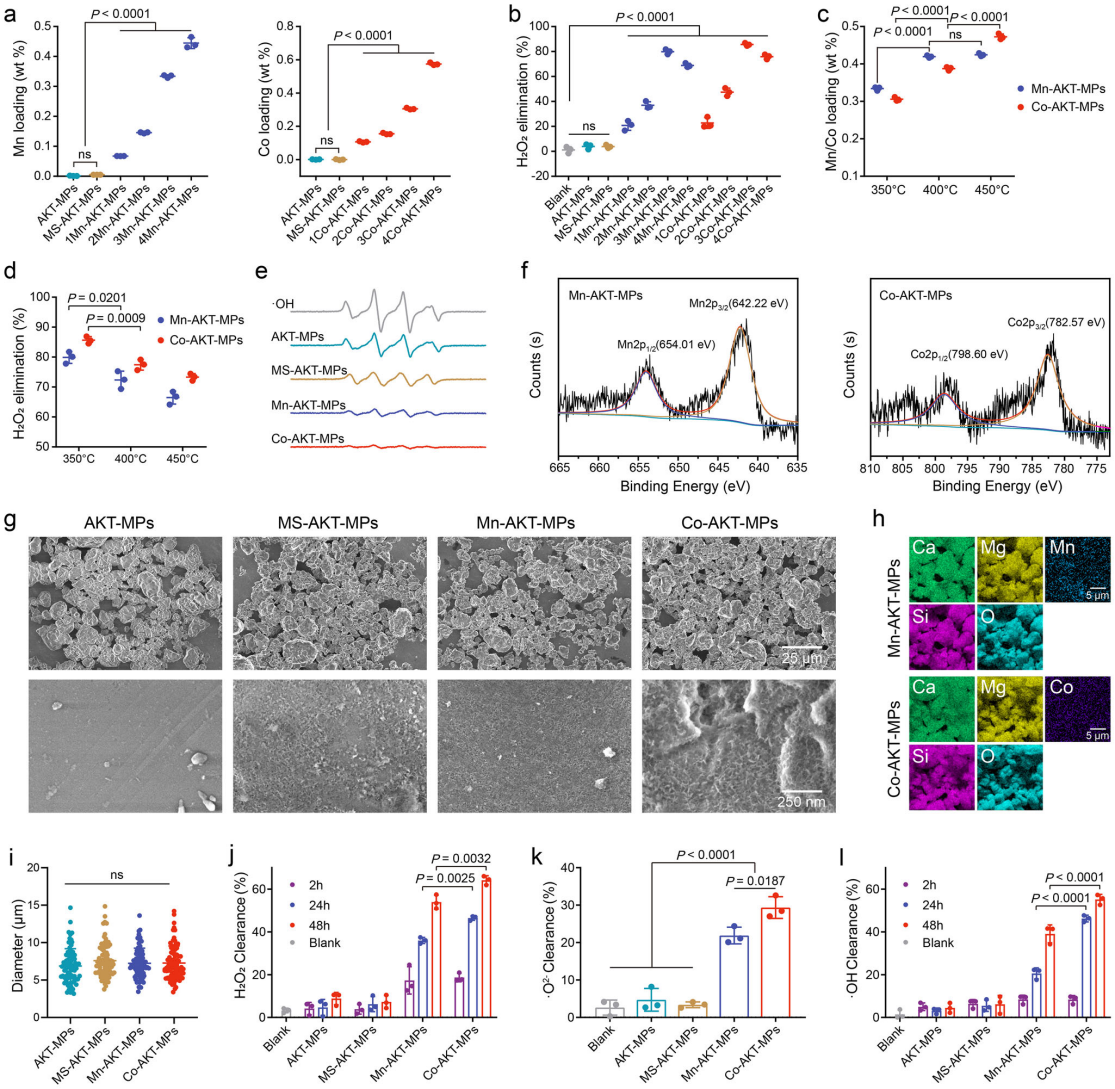
Construction and characterizations of Mn‐AKT‐MPs and Co‐AKT‐MPs. a) Quantification of manganese (Mn, left) or cobalt (Co, right) elements retained on Mn‐AKT‐MPs or Co‐AKT‐MPs with different additions of Mn or Co content at 350 °C (*n* = 3). b) H_2_O_2_ scavenging activity of Mn‐AKT‐MPs or Co‐AKT‐MPs with different additions of Mn or Co content at 350 °C (*n* = 3). c) Quantification of Mn or Co elements retained on Mn‐AKT‐MPs or Co‐AKT‐MPs at various temperature (350, 400, and 450 °C; *n* = 3). d) H_2_O_2_ scavenging activity of Mn‐AKT‐MPs or Co‐AKT‐MPs with the same additions of Mn or Co content at various temperature (*n* = 3). e) ESR spectra of the different formulations using DMPO as a trapping agent. f) XPS characteristic analysis of Mn 2p or Co 2p in Mn‐AKT‐MPs or Co‐AKT‐MPs, respectively. g) Representative SEM image of AKT‐MPs, MS‐AKT‐MPs, Mn‐AKT‐MPs, and Co‐AKT‐MPs. Bottom: surface morphology of MPs under high magnification. h) Elemental mapping of calcium (Ca, green), magnesium (Mg, yellow), silicon (Si, purple), oxygen (O, teal), Mn (distant blue), and Co (indigo) in Mn‐AKT‐MPs or Co‐AKT‐MPs. i) Average diameter of different formulations. *n* = 80 individual MPs. j–l) ROS scavenging activity of Mn‐AKT‐MPs and Co‐AKT‐MPs at 0.15 mg/mL mimic catalase (CAT) j), SOD (K) and eliminate ·OH l). Data are presented as the mean ± SD. *p‐*values are shown in the graphs. Statistical significance was analyzed by one‐way ANOVA with Tukey's multiple comparisons test for (a), (b), (c), (d), (i), and (l), and by two‐way ANOVA with Tukey's multiple comparisons test for (j) and (k).

The synthesis procedure for decorating atomic Mn or Co catalysts on AKT microparticles via the molten salt method is shown in Scheme 1a. The mixture of salt (LiCl and KCl), AKT microparticles, and the precursor of Mn (MnCl_2_) or Co (CoCl_2_·6H_2_O) was heated in a semi‐capped corundum crucible to reach the melting point of the salts, resulting in the formation of a liquid environment that homogeneously disperses Mn^2+^ or Co^2+^ ions on the AKT microparticles. Following a 1‐h exposure to a fusion temperature of 350 °C under Ar flow and subsequent natural cooling, the sample was thoroughly washed with water to remove residual salt. The amount of Mn or Co loaded on the AKT microparticles was found to increase with increasing the addition of the precursors, as detected by inductively coupled plasma atomic emission spectrometry (ICP‐AES) (**Figure** **1**a; Table S1, Supporting Information). The H_2_O_2_ scavenging rate of Mn‐AKT‐MPs increases from 20.74 ± 3.89% to 79.88 ± 1.98% when the loading amount of Mn varies from 0.067 to 0.334 wt.%. However, further enhancing the Mn content to 0.445 wt.% leads to an obvious decrease in activity, which could be attributed to the saturation of surface‐active sites with a relatively high loading amount (Figure 1b). Regarding the Co‐AKT‐MPs, there is a similar pattern of change. With the increase of Co content to 0.306 wt.%, the H_2_O_2_ elimination rate reached 85.63 ± 1.18%, which is higher than that of Mn‐AKT‐MPs, and then the catalytic rate decreased with the further increase of Co loading (Figure 1b). To investigate the influence of the operating temperature on the catalytic performance, we tested the loading amount of Mn or Co at various treatment temperatures (350, 400, and 450 °C), with a constant precursor (MnCl_2_ or CoCl_2_·6H_2_O) was added. It was found that both Mn‐AKT‐MPs and Co‐AKT‐MPs showed an increase in the loading of Mn or Co species with temperature increase (Figure 1c). However, the catalytic activity gradually decreased when the process temperature was increased above the melting point of the salts, manifesting that the treatment temperature of 350 °C is optimal for the construction of antioxidant MPs (Figure 1d). The amount of O_2_ produced by the decomposition of H_2_O_2_ was also monitored to assess the catalytic activity of various MPs. Similar results were observed that the treatment temperature and loading amount of Mn or Co species affected the production of O_2_ significantly (Figure S1 and S2, Supporting Information). Consequently, we developed antioxidant MPs by decorating 0.334 wt% Mn or 0.306 wt% Co at a treatment temperature of 350 °C for the subsequent assays due to the superior catalytic activity. Using 5,5′‐dimethylpyrroline‐1‐oxide (DMPO) as a spin‐trapping agent, electron spin resonance (ESR) spectroscopy further confirmed the ·OH scavenging effect of Mn‐AKT‐MPs and Co‐AKT‐MPs, where the 1:2:2:1 multiple peak in the ESR spectrum indicated the presence of ·OH in the AKT‐MPs or MS‐AKT‐MPs group, and the peaks were greatly reduced upon the addition of Mn‐AKT‐MPs and even more upon the treatment of Co‐AKT‐MPs (Figure 1e). To investigate the sustained catalytic activity, the capacity of different MPs to scavenge H_2_O_2_ over several consecutive cycles was evaluated (Figure S3, Supporting Information). The results revealed that Mn‐AKT‐MPs exhibited a slight reduction in H_2_O_2_ scavenging capability by the fourth cycle, decreasing from 82.91 ± 3.53% to 61.25 ± 6.54%. However, Mn‐AKT‐MPs still retained a robust ability to remove H_2_O_2_ even in the fourth cycle. On the other hand, Co‐AKT‐MPs demonstrated a modest decline in H_2_O_2_ scavenging efficiency across the four cycles. Importantly, there was no statistically significant difference observed, indicating the sustained ROS scavenging capacity of Co‐AKT‐MPs across repeated cycles.

The X‐ray diffraction (XRD) results demonstrate that the phase did not change before and after the molten‐salt treatment for AKT‐MPs, and all MPs still maintained high‐quality crystallinity (Figure S4, Supporting Information). The elemental peaks of Mn2p and Co2p were detected by X‐ray photoelectron spectroscopy (XPS) for the Mn‐AKT‐MPs and Co‐AKT‐MPs, respectively, suggesting that the Mn or Co could be decorated onto AKT‐MPs by a molten‐salt treatment (Figure S5, Supporting Information). In particular, the Mn 2p and Co 2p regions of XPS for Mn‐AKT‐MPs and Co‐AKT‐MPs are shown in Figure 1f. It was observed that the peaks of Mn 2p at ≈654.01 and 642.22 eV are assigned to Mn 2p_1/2_ and Mn 2p_3/2_, respectively, and Co 2p_1/2_ and Co 2p_3/2_ are located at 798.60 and 782.57 eV, respectively, indicating that the valence state of both Mn and Co is +2. Because transition metal ions with low‐valence state have strong electron‐donating ability, which contributes to high antioxidant activity to mimic SOD and CAT for the Mn‐AKT‐MPs or Co‐AKT‐MPs. Morphological analysis, performed by scanning electron microscopy (SEM, Figure 1g), revealed negligible change in the dimensions of MPs after the molten‐salt treatment, maintaining an approximate particle size of 7 µm (Figure 1i). However, a distinct change of surface morphology was observed between the untreated AKT‐MPs and molten‐salt treated MPs (MS‐AKT‐MPs, Mn‐AKT‐MPs, and Co‐AKT‐MPs), as the latter exhibited a rough exterior with velvety nanostructures (Figure 1g). Such surface metamorphosis is reasonably attributed to the well‐documented etching propensity inherent in the molten salt system.^[^ ^31^ ^]^ Furthermore, SEM together with energy dispersive X‐ray spectroscopy (EDX) elemental mapping showed the uniform distributions of Mn and Co elements on the surface of Mn‐AKT‐MPs and Co‐AKT‐MPs, respectively, consistent with the results of XPS, further confirming the successful loading of Mn and Co ions.

### 2.2 Cellular Evaluation of Mn‐AKT‐MPs and Co‐AKT‐MPs

Prior to exploring the potential therapeutic efficacy for OA, we first examined the potential cytotoxicity of Mn‐AKT‐MPs and Co‐AKT‐MPs in vitro, using RAW 264.7 and ATDC5 cells as model cell lines to represent immune cells and chondrocytes, respectively. Cell viability was assessed using Cell Counting Kit‐8 (CCK‐8) assays, which revealed no significant reduction in cell viability after treatment with Mn‐AKT‐MPs or Co‐AKT‐MPs at concentrations up to 0.15 mg mL^−1^ in both cell lines, indicating good biocompatibility (Figure S6a,b, Supporting Information). To confirm this finding, live/dead cell staining and imaging were performed after 24 h of incubation with different formulations at 0.15 mg mL^−1^. The results were consistent with the CCK‐8 assay and indicated equivalent cell viability to the control group (Figure S6c,d, Supporting Information). As a result, a concentration of 0.15 mg mL^−1^ was chosen for all subsequent in vitro experiments to evaluate the biological effects of various MPs.

**Figure 2:**
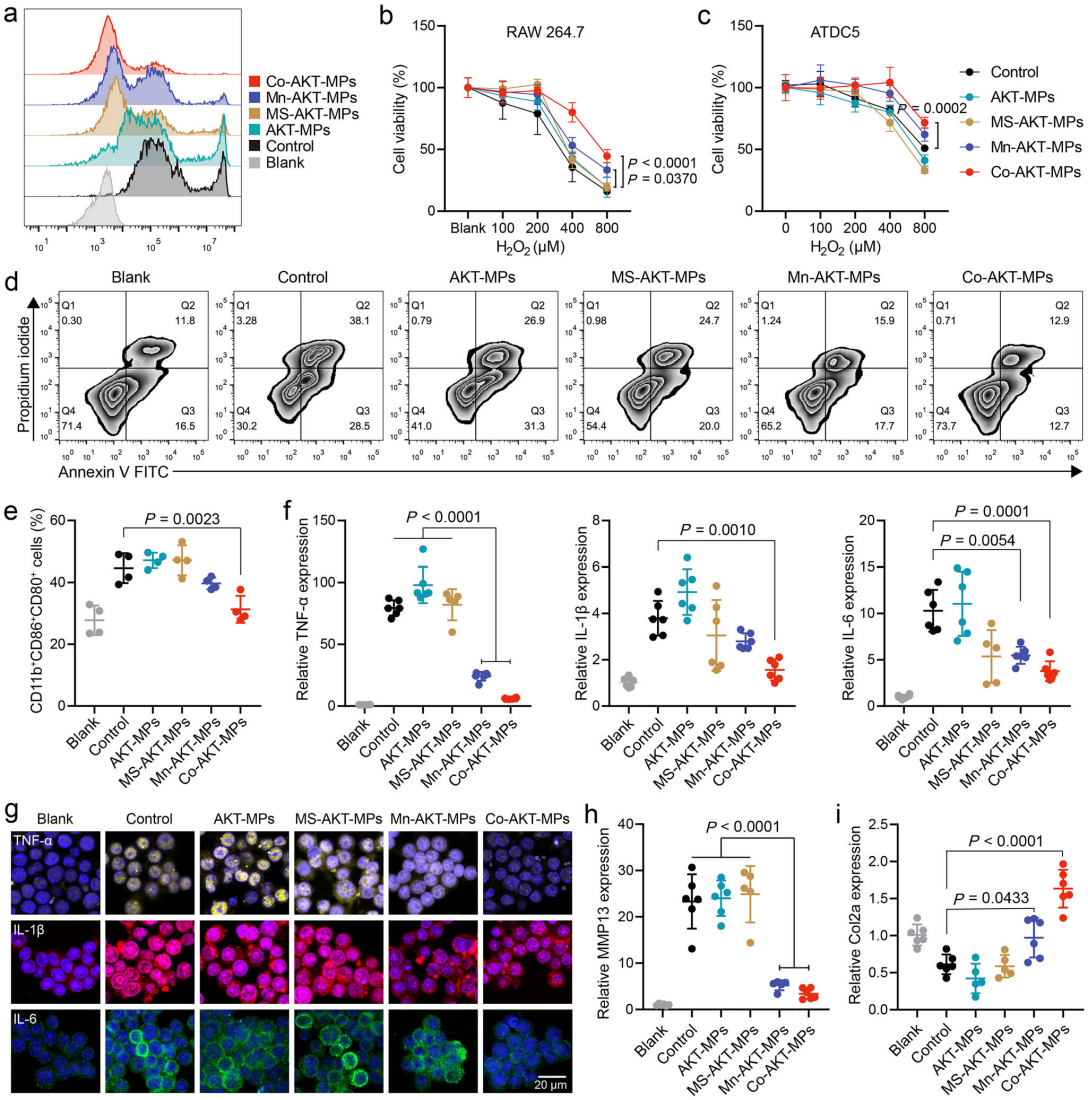
a) DCF fluorescence intensity in RAW 264.7 cells measured by flow cytometry after a 2 h stimulation with 200 µM H_2_O_2_ and simultaneous incubation with PBS control, AKT‐MPs, MS‐AKT‐MPs, Mn‐AKT‐MPs, or Co‐AKT‐MPs. The concentration of the different formulations was 150 µg mL^−1^. b,c) Cell viability of RAW 264.7 b) and ATDC5 c) treated with indicated microparticle formulations and exposed to different doses of H_2_O_2_ from 100 to 800 µM for 12 h (*n* = 5). d) Flow cytometric analysis based on Annexin V‐FITC/PI apoptosis staining after incubation with different treatments and being stimulated by 200 µM H_2_O_2_ (*n* = 3). e) Quantification of flow cytometry data of CD80^+^CD86^+^ cells gating on CD11b^+^ cells (*n* = 4). f) The relative gene expression of TNF‐α, IL‐1β, and IL‐6 was measured by quantitative real‐time polymerase chain reaction (RT‐PCR) in the blank, PBS‐, AKT‐MPs‐, MS‐AKT‐MPs‐, Mn‐AKT‐MPs‐, and Co‐AKT‐MPs‐treated H_2_O_2_‐stimulated RAW 264.7 cells (*n* = 6). g) Representative confocal microscopy images of cellular TNF‐α, IL‐1β, and IL‐6 in H_2_O_2_‐stimulated RAW 264.7 cells following various treatments. h,i) The relative gene expression of MMP13 h) and COL‐2a i) in H_2_O_2_‐stimulated ATDC5 cells after the above formulation treatments (*n* = 6). Data are presented as the mean ± SD. *p‐*values are shown in the graphs. Statistical significance was analyzed by two‐way ANOVA with Tukey's multiple comparisons test for (b) and (c), and by one‐way ANOVA with Tukey's multiple comparisons test for (e and f) and (h and i).

Next, the antioxidant activity of different MPs was measured at a concentration of 0.15 mg mL^−1^. The conducted assays confirmed the highly sensitive and time‐dependent scavenging of H_2_O_2_, ·OH, and O_2_·^−^ by Mn‐AKT‐MPs or Co‐AKT‐MPs. Notably, Co‐AKT‐MPs exhibited a higher level of antioxidant potency in comparison to Mn‐AKT‐MPs at this concentration (Figure 1j–l). We then evaluated the efficacy of various MPs as micro‐antioxidant in scavenging cellular ROS. Under 200 µM H_2_O_2_‐induced stress conditions, we observed an increase in intracellular ROS production in both RAW 264.7 and ATDC5 cells, as indicated by elevated levels of 2′,7′‐dichlorofluorescein (DCF) staining. The increased DCF staining could be significantly reduced by Mn‐AKT‐MPs and Co‐AKT‐MPs, but not by the control AKT‐MPs and MS‐AKT‐MPs (Figure S7, Supporting Information). Notably, the Co‐AKT‐MPs group exhibited a relatively lower cellular ROS level than the Mn‐AKT‐MPs group, which was even undetectable by confocal laser scanning microscopy (CLSM) imaging. Furthermore, quantitative flow cytometric analysis demonstrated the ROS‐scavenging effect of Mn‐AKT‐MPs and Co‐AKT‐MPs after co‐incubation with 200 µm H_2_O_2_‐stimulated RAW 264.7 cells for 2 h (**Figure** **2**a). These findings were further supported by observations of protected cell viability in both cell types and anti‐apoptotic ability in RAW 264.7 cells (Figure 2b–d; Figure S8, Supporting Information).

The pathogenesis of OA is closely linked to the infiltration of pro‐inflammatory cells into the synovium and synovial fluid, particularly macrophages, which comprise 12–40% of the synovial immune cells and are also the major leukocyte population in the synovial fluid. Pro‐inflammatory macrophages are generated in response to multiple biological cues, including excessive ROS, resulting in a cascade of events that contribute to the progression of the disease.^[^ ^6b^ ^]^ To investigate whether the various MPs could alleviate the inflammatory response of macrophages, we incubated the murine mononuclear macrophage cell line RAW 264.7 with H_2_O_2_ stimulation and were treated with PBS or 0.15 mg mL^−1^ of various MPs for 12 h. The cells were collected, and markers of M1 macrophages were detected through flow cytometry. As shown in Figure 2e and Figure S9 (Supporting Information), Co‐AKT‐MPs sharply downregulated the costimulatory molecules of CD80 and CD86 when compared with the groups of control, AKT‐MPs, MS‐AKT‐MPs, and Mn‐AKT‐MPs. Despite a declining trend observed in the Mn‐AKT‐MPs group in comparison to the control group, the difference between them was not statistically significant, which suggests that the Co‐AKT‐MPs was more effective than Mn‐AKT‐MPs in reversing the macrophage phenotype. Activated M1 macrophages secrete several pro‐inflammatory cytokines, including tumor necrosis factor‐α (TNF‐α), interleukin‐1β (IL‐1β), and interleukin‐6 (IL‐6), which negatively affect the normal activity of chondrocytes and mesenchymal cells.^[^ ^12^, ^32^ ^]^ Next, we detected the expression of these mRNA in H_2_O_2_‐stimulated RAW 264.7 cells by quantitative real‐time polymerase chain reaction (RT‐PCR). When compared with the control group, the cells treated with Mn‐AKT‐MPs exhibited a significant decrease in the mRNA levels of TNF‐α (3.29‐fold) and IL‐6 (1.88‐fold). As for the Co‐AKT‐MPs group, all three cytokines were more effectively downregulated, with TNF‐α, IL‐1β, and IL‐6 mRNA levels being reduced by 13.12‐, 2.42‐, and 2.72‐fold, respectively (Figure 2f). To further confirm the ability of Mn‐AKT‐MPs and Co‐AKT‐MPs to inhibit macrophage polarization, we performed immunofluorescence staining to visualize the intracellular intensities of these pro‐inflammatory cytokines. As shown in Figure 2g, the amounts of all three cytokines in cells were more significantly reduced by treatment with Co‐AKT‐MPs than by treatment with Mn‐AKT‐MPs, consistent with the mRNA levels in Figure 2f. Overall, these findings indicate that Co‐AKT‐MPs effectively suppress the trend of polarization into the M1 phenotype of ROS‐exposed macrophages, whereas Mn‐AKT‐MPs do exhibit some anti‐inflammatory properties, but with less potency than Co‐AKT‐MPs.

Chondrocytes, the only cell type present in articular cartilage, are the major cellular targets for cartilage degradation in OA. This degradation is characterized by a catabolic phenotype that involves the excessive production of cartilage extracellular matrix (ECM)‐degrading proteases, including MMP13, as well as an anabolic inhibitory phenotype that results in a reduction in the production of ECM, particularly type II collagen (COL‐2). These processes play a crucial role in the initiation and progression of OA.^[^ ^3^, ^6^ ^]^ We therefore proceeded to investigate the ability of different MPs to protect the chondrocytes from oxidative pressure in vitro. To this end, we co‐cultured H_2_O_2_‐stimulated ATDC5 cells with different formulations for 12 h, followed by analysis of MMP13 and COL‐2a expressions. As illustrated in Figure 2h,i, compared with the control group, treatment with Co‐AKT‐MPs resulted in a large decrease in MMP13 expression (6.88‐fold) and an increase in COL‐2a transcription (2.68‐fold). It was noteworthy that Mn‐AKT‐MPs also showed a statistically significant difference in the protection of chondrocytes, with a 4.47‐fold reduction in MMP13 expression and a 1.59‐fold increase in COL‐2a expression relative to the control group. The above results indicate that both Mn‐AKT‐MPs and Co‐AKT‐MPs have potential as protective agents against oxidative stress‐induced macrophage and chondrocyte damage.

To assess the effect of MPs on the function of cell endogenous antioxidant enzymes. Various MPs were added to ATDC5 and RAW 264.7 cells under H_2_O_2_‐stimulated or nonstimulated conditions. Following a 12‐h incubation, we evaluated the relative mRNA expression of CAT and SOD3 in ATDC5 and RAW 264.7 cells (Figure S10, Supporting Information). The results revealed that in the absence of H_2_O_2_, the CAT expression in both ATDC5 and RAW 264.7 cells remained comparable to the blank group. However, under H_2_O_2_ stimulation, a significant increase in CAT expression was observed in groups treated with H_2_O_2_, AKT‐MPs, and MS‐AKT‐MPs, which may be a negative feedback mechanism triggered by oxidative stress. Notably, CAT expression in cells administered with Mn‐AKT‐MPs or Co‐AKT‐MPs resembled that of the blank group, reflecting their enhanced H_2_O_2_ scavenging capabilities. As for SOD3, a similar trend was observed in RAW 264.7 cells. However, a significant elevation in SOD3 expression was noted in ATDC5 cells, regardless of H_2_O_2_ exposure, which could suggest an inherent sensitivity of these chondrocytes to oxidative stimulation. Furthermore, Co‐AKT‐MPs treatment led to a notable reduction in SOD3 expression, highlighting its potent H_2_O_2_ scavenging ability.

As previous studies have shown that the gradient of H_2_O_2_ concentration between the extracellular and intracellular compartments is ≈100‐fold, and the latest genetically encoded H_2_O_2_ probes suggest that this gradient may be even higher, ranging from 200 to 650‐fold.^[^ ^33^ ^]^ We next explored whether MPs function intracellularly or extracellularly. After incubating ATDC5 and RAW 264.7 cells with Co‐AKT‐MPs for 12 h, we performed transmission electron microscopy (TEM) observations (Figure S11, Supporting Information). The results clearly showed the presence of small‐sized Co‐AKT‐MPs within the cytoplasm of both ATDC5 and RAW 264.7 cells. In contrast, relatively larger particles were found to have limited cellular uptake. This may be because we employed a physical grinding method to prepare the AKT‐MPs in this study. While the overall diameter of these MPs is in the micrometer range, there is indeed variability in the particle size distribution. Particles that are too large may not be internalized by cells. ROS was mainly generated within mitochondria inside cells, but was also distributed on cell membranes and in the extracellular space. Modulation of ROS levels at the cell membrane has also been shown to enhance cellular activity.^[^ ^34^ ^]^ Therefore, we speculate that MPs could scavenge ROS both intracellularly and extracellularly, and possibly even at the cell membranes.

### 2.3 Protective Efficacy in Cartilage Explants

**Figure 3:**
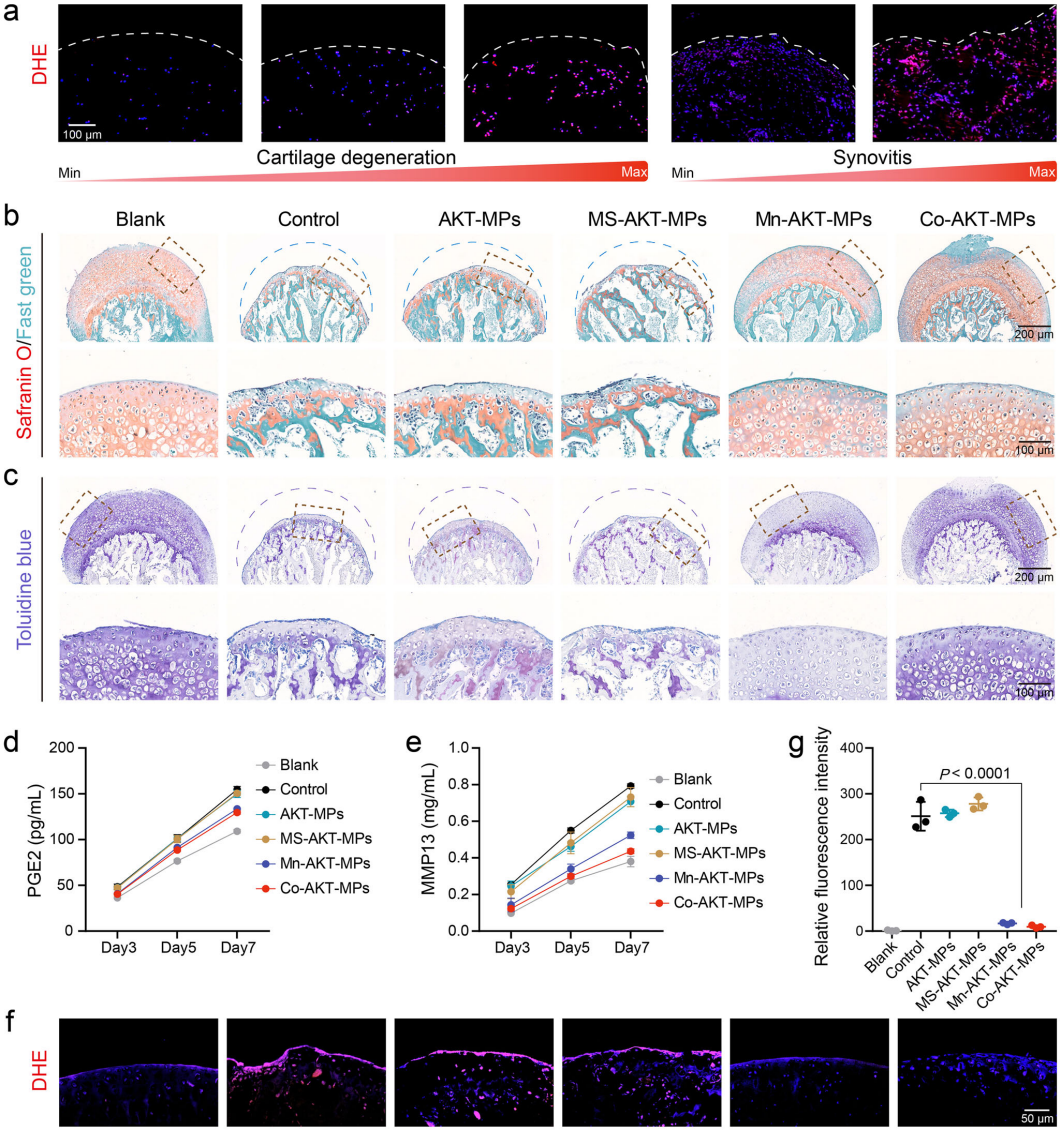
a) Representative images of DHE staining on the sections of cartilage and synovium tissues obtained at the human total arthroplasty of the knee joints. The intracellular ROS buildup increases in proportion to the severity of cartilage degeneration and synovitis. b) Safranin O/fast green staining of mouse femoral head explants under the indicated treatment conditions and continuous incubation with 200 µM H_2_O_2_ for 7 days. Bottom: Magnified images of the dashed boxed areas. c) Mouse femoral head sections were stained by toluidine blue staining for proteoglycans. Bottom: Magnified images of the dashed boxed areas. d,e) Effects of different formulations on PEG2 d) and MMP13 e) secretion at different time points, elicited by 200 µM H_2_O_2_ for 7 days. f,g) Representative fluorescence images f) and corresponding ROS intensities based on DHE staining g) from H_2_O_2_‐stimulated mouse femoral heads with or without various treatments (*n* = 3). Data are presented as the mean ± SD. *p‐*values are shown in the graphs. Statistical significance was analyzed by one‐way ANOVA with Tukey's multiple comparisons test.

To verify the elevated levels of oxidative stress in OA‐related tissues, human OA cartilage and synovial tissue samples obtained from surgical discards were stained with the ROS‐sensitive fluorescent dye dihydroethidium (DHE). In contrast to healthy human cartilage and synovial tissue, which exhibited minimal levels of ROS, significantly increased levels of ROS were detected in severely degenerated OA cartilage, and abundant staining was also observed in synovial tissue affected by synovial inflammation (**Figure** **3**a). The marked increase in ROS in OA cartilage and synovium suggests its potential role in OA development, as previously described,^[^ ^7^ ^]^ and supports the possibility of using Mn‐AKT‐MPs and Co‐AKT‐MPs to attenuate OA progression. The articular cartilage explants represent an in vitro model to study OA progression in a 3D environment. We harvested femoral heads from 12‐week‐old wild‐type mice and then stimulated them with 200 µM of H_2_O_2_. On days 0, 3, and 5, 150 µg mL^−1^ of different MPs were added, and the H_2_O_2_ was supplemented at each time. After 7‐day treatments, the AKT‐MPs and MS‐AKT‐MPs groups displayed similar cartilage damage, featured by severe ECM and chondrocyte loss. In contrast, Mn‐AKT‐MPs and Co‐AKT‐MPs blocked cartilage degeneration induced by H_2_O_2_, leading to comparable Safranin O and toluidine blue (TB) content as the blank group (Figure 3b,c; Figure S12, Supporting Information). Noteworthy, although Mn‐AKT‐MPs were able to significantly reverse the severe destruction of cartilage structure, there was still partial loss of proteoglycans as evidenced by TB staining (Figure 3c). Measurements of cumulative inflammatory factors, PGE2, and proteinases, MMP13, released into the supernatant further demonstrate that Co‐AKT‐MPs possess a better therapeutic effect than Mn‐AKT‐MPs (Figure 3d,e). Furthermore, DHE staining revealed a significant oxidative stress microenvironment in all control, AKT‐MPs, and MS‐AKT‐MPs groups, whereas little fluorescent signal was monitored in both Mn‐AKT‐MPs and Co‐AKT‐MPs groups (Figure 3f,g). Together, these data provide in vitro evidence that Mn‐AKT‐MPs and Co‐AKT‐MPs have protective action on cartilage against ROS‐inducing insults.

### 2.4 Anti‐Inflammation Effects In Vivo

**Figure 4:**
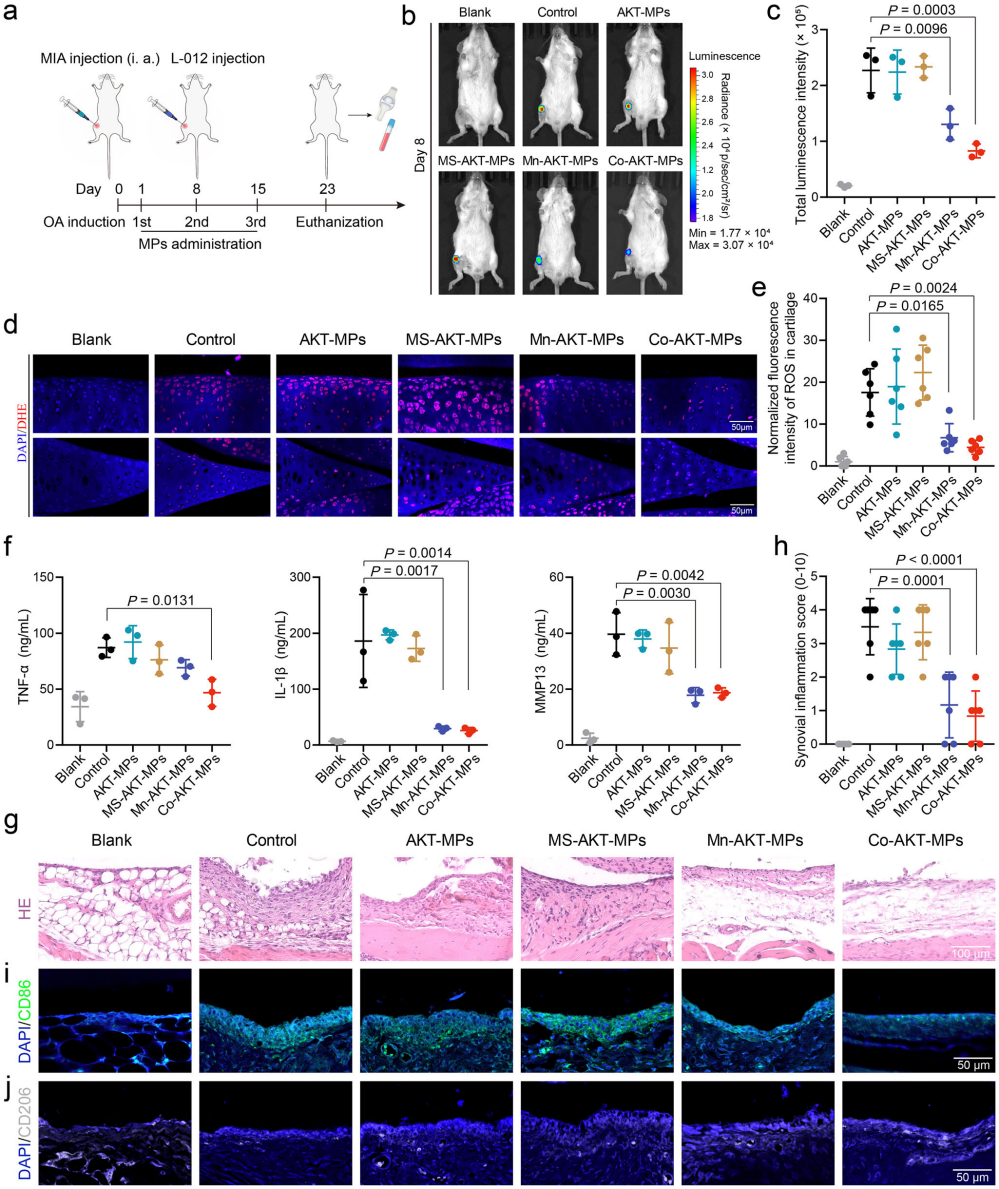
a) Schema showing the timeline of OA model construction and therapeutic regimen. BALB/c mice received an intra‐articular (i.v.) injection of monosodium iodoacetate (MIA) on day 0, then were treated with indicated MPs on days 1, 8, 15. L‐012 intensities of ROS were measured by the IVIS Spectrum system on day 8. Cartilage, subchondral bone, and synovial tissues pathology were analysed on day 23. b) Changes in ROS levels in the MIA‐stimulated knee joints on day 8 after OA induction and with various treatments were analysed using in vivo bioluminescence imaging. c) The luminescence intensities of L‐012, a luminol‐based chemiluminescent probe, were quantified (*n* = 3). d) Analysis of ROS levels of cartilage and menisci from OA mice on day 23 after the indicated engineered MPs treatments in vivo. ROS was revealed by DHE staining. e) Quantitative analysis of ROS intensities (*n* = 6). f) Levels of pro‐inflammatory cytokines of TNF‐α, IL‐1β, and matrix metalloproteinase‐13 (MMP13) in OA knee joints following different treatments (*n* = 3). g) Representative HE staining of synovium tissue on day 23 after OA induction. g) Synovial inflammation in OA knee joints was evaluated across indicated treatments (*n* = 6). i,j) Representative images of immunofluorescence staining for CD86 i) and CD206 j) on synovium tissue sections from mice treated in various groups. Data are presented as the mean ± SD. *p‐*values are shown in the graphs. Statistical significance was analyzed by one‐way ANOVA with Tukey's multiple comparisons test.

The feasibility of using Mn‐AKT‐MPs and Co‐AKT‐MPs to relieve joint inflammation and protect against cartilage destruction was assessed in mice with OA experimentally induced via monosodium iodoacetate (MIA) after intra‐articular injection. MIA injection triggers biochemical and histological pathologies that follow a biphasic pattern resembling human OA, with an early phase (0–7 days) marked by knee joint inflammation, excessive production of ROS and pro‐inflammatory cytokines (TNF‐α, IL‐1β, MMP13, etc), and a late phase (7–14 days) characterized by chondrocyte apoptosis, cartilage degradation, and ECM loss.^[^ ^35^ ^]^ Since previous work has reported a severe inflammatory response in the knee one day after MIA administration, we therefore randomly divided mice into five groups, including an untreated control group and four therapy groups (AKT‐MPs, MS‐AKT‐MPs, Mn‐AKT‐MPs, and Co‐AKT‐MPs), all of which received treatment on day 1 (**Figure** **4**a).

After 8 days of treatment, ROS levels in inflamed joints were noninvasively detected using a luminescent probe (L‐012) with an in vivo imaging system (IVIS). As shown in Figure 4b,c, the luminescence emitted from the joints of the untreated control group was significantly higher than that emitted from the healthy blank joints, confirming the MIA‐induced ROS overproduction. Treatment with AKT‐MPs or MS‐AKT‐MPs did not significantly reduce the intensity of the luminescent signal induced by MIA. In contrast, Mn‐AKT‐MPs and Co‐AKT‐MPs treatments showed a greater reduction in the detected luminescent intensity than other treatments. We then collected joints, and DHE staining showed that the proportion of positive chondrocytes was most decreased in both cartilage and meniscus after Co‐AKT‐MPs intervention (3.94‐fold versus control), followed by Mn‐AKT‐MPs (2.60‐fold versus control; Figure 4d,e). Collectively, in vivo data show that both Mn‐AKT‐MPs and Co‐AKT‐MPs possess the ability to scavenge high levels of ROS triggered by MIA, with Co‐AKT‐MPs exhibiting superior efficacy in comparison. Furthermore, after homogenizing the knee joints, enzyme‐linked immunosorbent assay (ELISA) for downstream inflammatory cytokines (that is, TNF‐α and IL‐1β) and proteases (MMP13) was conducted, which showed that treatment with Mn‐AKT‐MPs or Co‐AKT‐MPs resulted in a significant reduction of approximately 6‐fold in IL‐1β and 2‐fold in MMP13 levels, as compared to the control group (Figure 4f). However, in terms of TNF‐α, the Co‐AKT‐MPs treatment exhibited a noteworthy inhibitory effect (1.86‐fold versus control), whereas only a partial inhibition was observed in the case of Mn‐AKT‐MPs (Figure 4f).

Synovitis, which is characterized by hyperplasia of synovium and pro‐inflammatory immune cell infiltration, is a common feature of OA.^[^ ^36^ ^]^ The thickness of synovial tissue serves as an indicator of inflammation within the knee joint. PBS control‐treated inflamed knees displayed a significantly thickened synovium with more than threefold increase in the synovial inflammation score relative to sham knees. By contrast, inflamed knees treated with Mn‐AKT‐MPs and Co‐AKT‐MPs had only 1.17 and 0.83 values, respectively (Figure 4g,h). The presence of an inflammatory microenvironment is known to influence the phenotype of macrophages, which in turn can exacerbate the progression of OA.^[^ ^37^ ^]^ To further explore the immunomodulatory role of different MPs in vivo, immunofluorescence assays were performed to assess the phenotype of macrophages that had infiltrated into the synovium. Macrophages after Co‐AKT‐MPs intra‐articular injection were predominantly of the immunosuppressive CD206^+^ M2 phenotype, similar to those in the sham group, whereas the control, AKT‐MPs, and MS‐AKT‐MPs treatments had a significantly higher ratio of immune‐stimulatory CD86^+^ M1 phenotype macrophages (Figure 4i,j). Notably, the Mn‐AKT‐MPs only slightly altered the macrophage polarization in vivo, which was consistent with the in vitro data presented in Figure 2e. These results indicate that both Mn‐AKT‐MPs and Co‐AKT‐MPs possess the ability to scavenge ROS in vivo, suppress the expression of intra‐articular inflammatory factors and matrix‐degrading enzymes, and regulate macrophage phenotype. In particular, the overall anti‐inflammation efficacy of Co‐AKT‐MPs was found to be superior to that of Mn‐AKT‐MPs in all observed aspects of efficacy.

### 2.5 Alleviating the Progression of Cartilage Degeneration In Vivo

**Figure 5:**
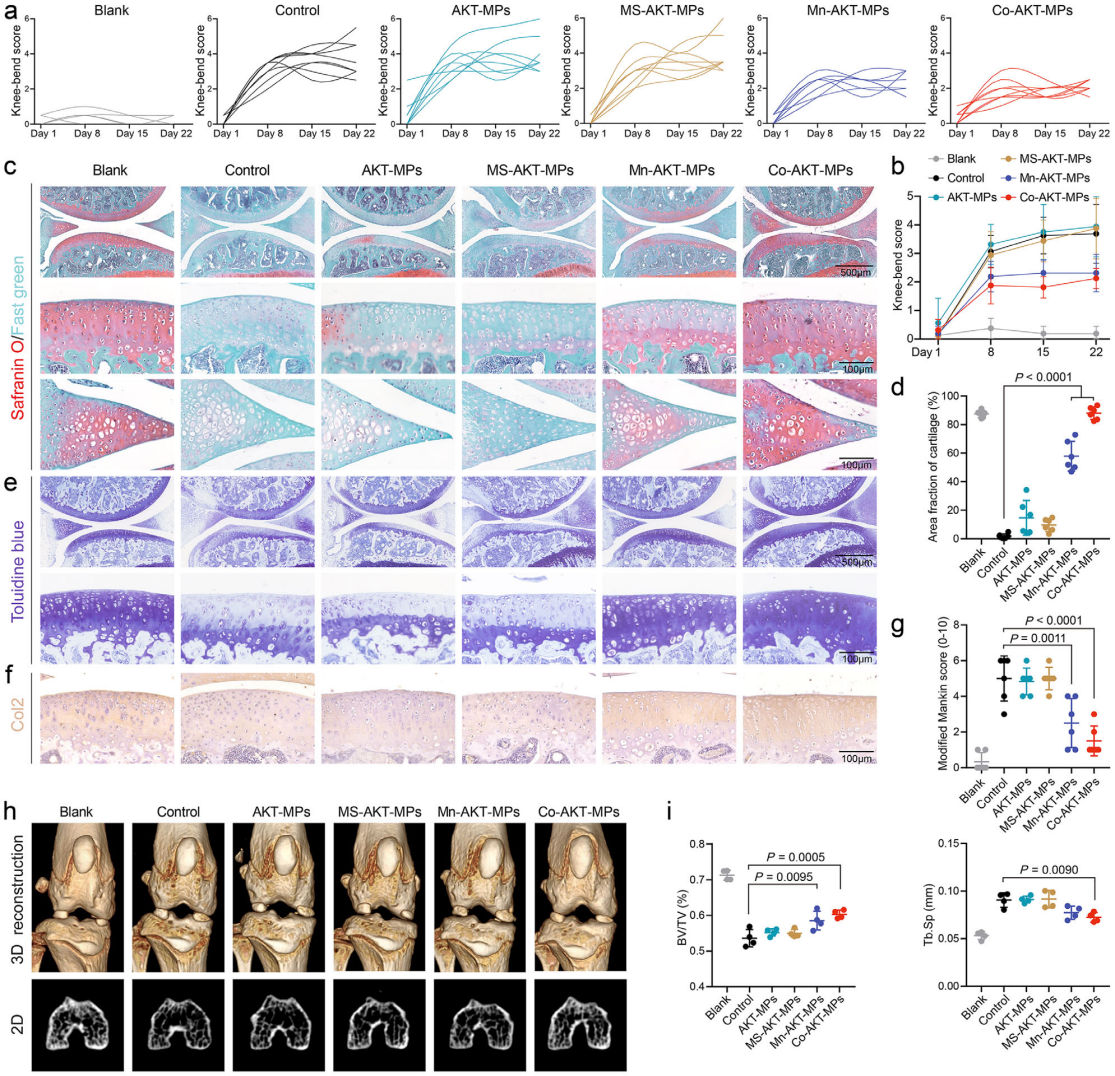
a) Knee‐bend scores (0–10) of each mouse after MIA injection following different treatments, recorded every week from day 1 to day 22 (*n* = 8). b) Curves of the average pain scores of MIA‐stimulated knee joints treated in various groups. c) Representative safranin O/fast green‐stained sagittal sections of the medial tibia, meniscus, and femur in knee joints treated in various groups. d) Quantification of safranin O‐positive area among the different groups (*n* = 6). e) Representative toluidine blue‐stained sagittal sections of the knee joints treated in various groups. f) Immunohistochemical staining of COL‐2 (brown) in the cartilage collected from various treated knee joints. g) Grading of OA severity according to modified Mankin scores on day 23 after OA induction (*n* = 6). h) Subchondral changes were analyzed by 3D (top) and 2D (bottom) micro‐CT images. i) The parameters of subchondral bone micro‐architecture were quantified (*n* = 4). BV/TV, ratio of bone volume to tissue volume; Tb.Sp, Trabecular separation. Data are presented as the mean ± SD. *p‐*values are shown in the graphs. Statistical significance was analyzed by one‐way ANOVA with Tukey's multiple comparisons test.

Encouraged by the unique anti‐inflammatory function of Mn‐AKT‐MPs and Co‐AKT‐MPs in MIA‐induced mice, we then further investigated the chondroprotection efficacy of various MPs against OA inflammation in its late phase. To test the sensitivity to the normal movement of each joint, a knee‐bend test of nociception for each mouse was performed throughout the treatments. There were no statistically significant differences in the knee scores among the control, AKT‐MPs, and MS‐AKT‐MPs animal groups, which were significantly different from the sham group from day 8 to day 22. Nevertheless, after finishing the therapeutics on day 22, the injection of Mn‐AKT‐MPs and Co‐AKT‐MPs resulted in lower values, with mean values decreasing from 3.69 ± 0.97 in the control group to 2.31 ± 0.61 and 2.13 ± 0.33 in the Mn‐AKT‐MPs and Co‐AKT‐MPs groups, respectively, implying that both Mn‐AKT‐MPs and Co‐AKT‐MPs are able to relieve OA pain (**Figure** **5**a,b).

Safranin O/Fast green staining, toluidine blue staining, immunohistochemistry, and computed tomography (micro‐CT) were carried out to evaluate the therapeutic effects of Mn‐AKT‐MPs and Co‐AKT‐MPs on cartilage degeneration at the end of the treatment. As shown in Figure 5c–f and Figures S13–S15 (Supporting Information), the proteoglycans and COL‐2 of articular cartilage in untreated control group were weakly expressed, indicating marked degeneration of cartilage ECM. Following three repeated injections of each test MPs at seven‐day intervals (day 1, day 8, and day 15), only the groups that received Mn‐AKT‐MPs or Co‐AKT‐MPs exhibited strong expression of ECM macromolecules, with an area fraction of cartilage reaching 57.90% and 88.02%, respectively, indicating arrested progression of OA in the knees (Figure 5d). In contrast, repeated treatments with saline, AKT‐MPs, or MS‐AKT‐MPs had almost no inhibitory effect on cartilage ECM degeneration, leading to the differences among the three groups were not significant in Mankin scores, whereas treatment with Mn‐AKT‐MPs or Co‐AKT‐MPs improved the Mankin scores to 2.50 ± 1.37 or 1.50 ± 0.83, respectively (Figure 5g). Interestingly, no difference was observed in the expression of COL‐1 and COL‐10 associated with the hypertrophic state of chondrocytes in all groups, which may be because intra‐articular injection of MIA was not sufficient to induce chondrocytes into a hypertrophic state in the absence of mechanical instability (Figure S16, Supporting Information). Apoptosis in cartilage and meniscus was further evaluated by TdT‐mediated dUTP nick‐end labeling (TUNEL) assay. It can be seen in the Figure S17 (Supporting Information), that the apoptotic rates of the chondrocytes are reduced after the treatment with Mn‐AKT‐MPs or Co‐AKT‐MPs, in contrast to those of MIA‐induced mice treated with AKT‐MPs or MS‐AKT‐MPs, respectively, well demonstrating the ability of Mn‐AKT‐MPs and Co‐AKT‐MPs in the prevention of MIA‐induced apoptosis.

To further evaluate the subchondral bone changes, micro‐CT scanning was performed with both 3D reconstruction and 2D images. As shown in Figure 5h, an unsmooth bone surface was observed in the control group, while AKT‐based antioxidants, especially Co‐AKT‐MPs, provided effective protection of subchondral bone with a smooth surface. Moreover, among the MPs‐treated groups, significant changes in bone volume/total volume (BV/TV), an indicator of changes in bone mass, were observed in both the Mn‐AKT‐MPs and Co‐AKT‐MPs groups (Figure 5i). However, Mn‐AKT‐MPs group was not significantly different from the control group in terms of trabecular separation (Tb.Sp), bone surface/bone volume ratio (BS/BV), and trabecular thickness (Tb.Th; Figure 5i; Figure S18, Supporting Information). It is noteworthy that trabecular number (Tb.N) and trabecular pattern factor (Tb.Pf) were no significant alterations among all groups (Figure S18, Supporting Information). This may be due to the MIA‐induced OA model being more suitable to reflect the features of early OA. Overall, these results indicated that Co‐AKT‐MPs could be more effective in maintaining bone mass and trabecular structure in a mouse model of early‐stage OA.

Before evaluating the potential toxicity of different MPs in mice, we first measured the ion release from different MPs in Tris‐HCl buffer (Figure S19, Supporting Information). The results indicate that after 1, 3, and 5 days of immersion, the cumulative release of Mn^2+^ from Mn‐AKT‐MPs was 4.0 ± 0.8 ppm, 6.4 ± 1.3 ppm, and 6.7 ± 1.2 ppm, respectively, while the Co^2+^ from Co‐AKT‐MPs were 3.5 ± 0.4, 4.9 ± 0.5, and 5.1 ± 0.5 ppm, respectively. These values are considerably lower than those reported to cause toxic effects.^[^ ^38^ ^]^ We then assessed potential in vivo toxic effects on serum biochemical and hematological parameters after serial intra‐articular injection of AKT‐MPs, MS‐AKT‐MPs, Mn‐AKT‐MPs, or Co‐AKT‐MPs in mice. As depicted in Figure S20 (Supporting Information), we did not identify any clinical signs of toxicity. Furthermore, we evaluated the histopathology of major organs such as the heart, liver, spleen, lung, and kidney of the treated mice and did not observe any histopathological abnormalities in any of the organs (Figure S21, Supporting information). In addition, we performed HE and TUNEL staining of cartilage and synovium from the mice without MIA induction, who were treated with various MPs, and did not detect any cell apoptosis (Figure S22, Supporting information). Taken together, these results suggest that various AKT‐based MPs, when administered at the adopted intra‐articular dosages, do not exhibit any discernible signs of toxicity in mice. Although the current safety study showed that the experimental concentration of the MPs was biocompatible to the experimental animals, in previous safety studies of orthopedic implants, the toxicity of wear ceramic particles and cobalt ions was still the main safety issues.^[^ ^39^ ^]^ Therefore, the biocompatibility of the MPs also needs further long‐term observation, and it is expected that the surfaces of orthopedic implants could also be modified by this method to reduce the loosening of prostheses caused by implant wear particles.

## 3 Conclusion

In summary, we synthesized the atomically distributed Mn or Co catalyst on the surface of AKT microparticles through a controlled molten‐salt method. The obtained Mn‐AKT‐MPs or Co‐AKT‐MPs exhibited broad‐spectrum ROS‐scavenging activities and satisfactory biocompatibility. By eliminating excessive ROS in an inflamed microenvironment, the administered Mn‐AKT‐MPs or Co‐AKT‐MPs demonstrated a notable capacity for protecting chondrocytes and preserving cartilage matrix by orchestrating homeostasis between anabolism and catabolism. Additionally, these microparticles exhibited a capability to impede the transition of macrophages toward a pro‐inflammatory M1 phenotype, thereby contributing to the amelioration of synovial inflammation. Moreover, the Mn‐AKT‐MPs or Co‐AKT‐MPs were also able to alleviate pain symptoms in mice with OA, restoring behavioral patterns similar to those of the sham group. Hence, such atomic catalyst‐integrated microparticles with high antioxidative capability show great potential for the treatment of OA, and the strategy proposed in this study may provide a new, long‐lasting, and comprehensive solution to neutralize excessive ROS caused by inflammatory diseases. The characterization of Mn‐AKT‐MPs and Co‐AKT‐MPs also demonstrates the potential of exploring the application of the molten‐salt method, a synthetic approach from catalytic chemistry, in the biomedical field.

## 4 Experimental Section

### Materials

Lithium chloride (LiCl, ≥ 98%), potassium chloride (KCl, 98%), cobalt(II) chloride hexahydrate (CoCl_2_·6H_2_O, ≥ 98.5%), nitric acid (HNO_3_, 69%), tris (hydroxymethyl) aminomethane (Tris), 5,5′‐dimethylpyrroline‐1‐oxide (DMPO), and hydrogen peroxide (H_2_O_2_, ≥ 30.0%) were purchased from Sinopharm Chemical Reagent (Shanghai, China). Manganous chloride (MnCl_2_, ≥ 99%) was obtained from Sigma‐Aldrich (Shanghai, China). Akermanite (Ca_2_MgSi_2_O_7_, AKT) was acquired from Kunshan Technology (Jiangsu, China). Catalase assay kit, SOD assay kit, DAPI, DCFH‐DA, and paraformaldehyde (PFA) were purchased from Beyotime (Shanghai, China). The hydroxyl radical antioxidant capacity (HORAC) assay kit was provided by Cell Biolabs (San Diego, USA). Dulbecco's modified Eagle's medium (DMEM) and fetal bovine serum (FBS) were purchased from LABLEAD (Beijing, China). Cell counting kit‐8 (CCK8), annexin V‐FITC and PI, Hifair II 1st Strand cDNA Synthesis Kit, and Hieff qPCR SYBR Green Master Mix were purchased from Yeasen (Shanghai, China). PE‐anti‐mouse CD11b (101 207, clone name: M1/70, 1:100), PE/Cy7‐antimouse CD80 (104 734, clone name: 16‐10A1, 1:100), and APC‐anti‐mouse CD86 (105 012, clone name: GL−1, 1:100) antibodies for flow cytometry analysis were obtained from BioLegend (San Diego, USA). Anti‐TNF‐α antibody (ab183218, clone name: EPR19147, 1:5000), anti‐IL‐1β antibody (ab254360, clone name: EPR23851‐127, 1:100), anti‐IL‐6 antibody (ab179570, clone name: EPR16610‐69, 1:100), secondary antibody conjugated with Alexa Fluor 488, and secondary antibody conjugated with Alexa Fluor 594 were purchased from Abcam (Cambridge, USA). Anti‐COL‐2 antibody (MA5‐13026, clone name: 6B3, 1:1000) and TRIzol Reagent were obtained from Invitrogen (Carlsbad, USA). Anti‐COL‐1 antibody (14695‐1‐AP, polyclonal antibody, 1:1000) was acquired from Proteintech (Wuhan, China). Anti‐COL‐10 antibody (GTX37732, polyclonal antibody, 1:500) was acquired from GeneTex (Irvine, USA). TNF‐α, IL‐1β, and IL‐6 enzyme‐linked immunosorbent assay (ELISA) kits were purchased from BioLegend (San Diego, USA). PGE2 ELISA kit was provided by Dogesce (Beijing, China). MMP13 ELISA kit was obtained from Cloud‐Clone Corp (Houston, USA). Dihydroethidium (DHE) was purchased from Harveybio (Beijing, China). Safranin‐O/fast green and toluidine blue stain solutions were obtained from Solarbio Life Science (Beijing, China).

### Preparation of Mn‐AKT‐MPs and Co‐AKT‐MPs

The molten‐salt method was used to prepare Mn‐AKT‐MPs and Co‐AKT‐MPs, and KCl‐LiCl was employed as the molten salt system. The mass ratio of KCl: LiCl was 1.1: 0.9, which can form a molten state at 350 °C and above, thus, 350 °C was chosen as the lowest temperature for the molten‐salt treatment. Briefly, 3.0 g AKT‐MPs, 3.3 g KCl, and 2.7 g LiCl were mixed with 7.5, 15.0, 30.0, or 60.0 mg of MnCl_2_, respectively, and they were ground in an agate mortar. After transfer to a crucible, the mixture was placed in a furnace, followed by heating to 350 °C at a rate of 5 °C min^−1^ under an argon atmosphere for 1 h. After cooling to room temperature, the crucible was removed, and the mixture was washed five times with deionized water to remove the soluble salts, and the washed powders were dried in an oven at 60 °C for 24 h. The same procedure was used to prepare Co‐AKT‐MPs, in which 3.3 g KCl, and 2.7 g LiCl were mixed with 14.0, 28.0, 56.0 or 112.0 mg CoCl_2_·6H_2_O, respectively. Mn‐AKT‐MPs prepared with different Mn additions were named 1Mn‐AKT‐MPs, 2Mn‐AKT‐MPs, 3Mn‐AKT‐MPs, and 4Mn‐AKT‐MPs. The Co‐AKT‐MPs prepared with different cobalt salt additions were named as 1Co‐AKT‐MPs, 2Co‐AKT‐MPs, 3Co‐AKT‐MPs, and 4Co‐AKT‐MPs. When no MnCl_2_ or CoCl_2_·6H_2_O was added, the MPs prepared by the molten‐salt treatment were named as MS‐AKT‐MPs. Additionally, the same method was used to prepare Mn‐AKT‐MPs and Co‐AKT‐MPs at three different molten‐salt treatment temperatures (350, 400, or 450 °C) while maintaining the same amount of manganese or cobalt salt additions (3.0 g AKT‐MPs with 30.0 mg of MnCl_2_ or 56.0 mg of CoCl_2_·6H_2_O).

### Characterization of Mn‐AKT‐MPs and Co‐AKT‐MPs

Mn or Co contents in Mn‐AKT‐MPs or Co‐AKT‐MPs and time‐dependent ion release kinetics were detected by inductively coupled plasma atomic emission spectrometry (ICP‐AES, Vista AX, USA). All the experiments were repeated three times. X‐ray photoelectron spectroscopy (XPS) spectrum and XPS characteristic spectrum for Mn 2p or Co 2p were obtained on ESCAlab250 (Thermo Fisher Scientific, USA). The morphology, element mappings, and size distribution of various MPs were characterized using scanning electron microscopy (SEM, S‐4800, Hitachi, Japan). X‐ray diffraction (XRD) patterns were recorded on a D8 Advance diffractometer (Bruker, Germany).

### Enzyme‐Like Activities

Antioxidation was evaluated by electron spin resonance (ESR) spectroscopy. The Fenton reaction (10 mM FeSO_4_ and 10 mM H_2_O_2_) was applied to produce hydroxyl radicals. In an ESR spectrum (Bruker EMX1598 spectrometer), DMPO was selected as the spin‐trapping agent to measure the effect of Mn‐AKT‐MPs and Co‐AKT‐MPs (10 mg mL^−1^) in removing hydroxyl radicals. In the ESR spectra of the DMPO‐OH• adducts, the change of the relative peak intensity indicated the ROS scavenging effect of various MPs.

The three main ROS, O_2_^•−^, H_2_O_2_, and •OH, were applied to determine the ROS scavenging capability of Mn‐AKT‐MPs and Co‐AKT‐MPs. Different assay protocols were followed in all experiments. The hydrogen peroxide quenching activity was conducted with the catalase assay kit. HORAC assay kits were used to measure hydroxyl radical scavenging activity. The superoxide anion scavenging activity was performed with a SOD assay kit.

### Animals and Cells

Male BALB/c mice (12 weeks old) were obtained from SPF Biotechnology. All animal experiments were approved by the Animal Care and Use Committee of Peking University, and the methods in the current work were complied with the Guide for the Care and Use of Laboratory Animals published by the National Academy Press (National Institutes of Health Publication No. 85‐23, revised 1996). The RAW 267.4 macrophage cells and chondrogenic cell line ATDC5 were cultured in DMEM medium containing 10% FBS, 100 U/mL penicillin G sodium, and 100 µg mL^−1^ streptomycin. The cells were cultured at 37 °C in a humidified atmosphere with 5% CO_2_.

### Cell Viability

The cytotoxicity of AKT‐MPs, MS‐AKT‐MPs, Mn‐AKT‐MPs, and Co‐AKT‐MPs was determined by a CCK‐8 assay. Briefly, RAW 264.7 and ATDC5 cells were seeded in 96‐well plates overnight at a density of 8000 cells per well. Then, the MPs with various concentrations from 39 to 5000 µg mL^−1^ were added and incubated for 24 h. The CCK‐8 assay was used to determine the viability of RAW 264.7 and ATDC5 cells after various treatments. The living cells assay against RAW 264.7 and ATDC5 cells was observed by confocal laser scanning microscope (Zeiss 710, Zeiss Microsystems) after Calcein‐AM and PI double staining. Briefly, RAW 264.7 and ATDC5 cells were incubated with 150 and 300 µg mL^−1^ different MPs for 24 h, followed by Calcein‐AM and PI staining in PBS for 30 min and washed three times with PBS before observation. To test the protective effect of Mn‐AKT‐MPs and Co‐AKT‐MPs, RAW 264.7 and ATDC5 cells were seeded in 96‐well plates overnight at a density of 8000 cells per well. Then, cells were incubated with 150 µg mL^−1^ different MPs in the presence of various concentrations of H_2_O_2_ ranging from 100 to 800 µM. After 24 h incubation, CCK‐8 assay was performed to measure cell viability. Cell culture without the addition of MPs or H_2_O_2_ was included as an essential control. To demonstrate the anti‐apoptotic effect of Mn‐AKT‐MPs and Co‐AKT‐MPs, RAW 264.7 cells were seeded in six‐well plates, then incubated with 150 µg mL^−1^ different MPs and stimulated with 200 µM H_2_O_2_ for 24 h. After different treatments, cells were collected and stained with annexin V‐FITC and PI. Finally, cells were washed with PBS three times and analyzed using flow cytometry.

### Intracellular ROS Measurement

RAW 264.7 and ATDC5 cells were seeded in 6‐well plates overnight, followed by the addition of 150 µg mL^−1^ different MPs and 200 µM H_2_O_2_. After 4 h incubation, intracellular ROS was stained by a fluorescence probe 2′,7′‐dichlorofluorescein diacetate (DCFH‐DA) according to the manufacturer's protocol. Finally, intracellular ROS was observed by confocal laser scanning microscopy and quantitatively analyzed by flow cytometry.

### In Vitro Macrophage Polarization Assay

Six well plates were seeded with RAW 264.7 cells at a density of 1 × 10^5^ cells per well, and cells were allowed to reach 50% subconfluency after 12 h incubation. Following the stimulation with 200 µM H_2_O_2_, the cells were treated with 0.15 mg mL^−1^ of various MPs for 12 h. After the treatments were completed, the medium was aspirated, and the cells were collected after washing with PBS. The cells were then stained with anti‐CD11b, anti‐CD80, and anti‐CD86 antibodies and analyzed by flow cytometry. FlowJo software was used to analyze the results.

### Gene expression Analysis

RAW 264.7 and ATDC5 cells were seeded in 6‐well plates overnight. After incubation with 150 µg mL^−1^ different MPs and 200 µM H_2_O_2_ for 24 h, cells were harvested, and total RNA was extracted using TRIzol reagent. Hifair II 1st Strand cDNA Synthesis Kit was used to reverse‐transcribe mRNA into complementary DNA (cDNA). Following this, Hieff qPCR SYBR Green Master Mix was used to perform polymerase chain reaction (PCR). Primers for TNF‐α, IL‐1β, IL‐6, MMP13, and COL‐2a were used, and the primer sequences for genes are listed in Table S2 (Supporting Information). GAPDH expression was served as an internal control. The relative expression of the genes was evaluated by the ΔΔC_t_ method.

### Immunofluorescence

To visualize the expressions of cellular pro‐inflammatory cytokines TNF‐α, IL‐1β, IL‐6, H_2_O_2_‐stimulated RAW 264.7 cells after different treatments were fixed in 4% paraformaldehyde and permeabilized. The fixed cells were blocked with 10% (v/v) fetal bovine serum at 37 °C for 1 h and then incubated with antibodies against IL‐1β, IL‐6, or TNF‐α overnight at 4 °C, followed by Alexa Fluor 488 or 594 goat anti‐rabbit IgG antibody for 1 h at room temperature. The nuclei were counterstained with DAPI. The samples were viewed by a confocal laser scanning microscope (Zeiss 710, Zeiss Microsystems). For synovium tissues immunofluorescence, samples were harvested and rapidly embedded in optimal cutting temperature (OCT) compound. Next, they were cut into sections (8 µm) and washed with PBS to remove OCT compound. The sections were then incubated with DHE for 30 min at 37 °C, followed by three washes with PBS. The nuclei were also counterstained with DAPI. Finally, the photomicrographs were obtained using a confocal laser scanning microscope.

### Human Articular Cartilage and Synovium Samples

A total of three OA patients hospitalized for total joint replacement surgeries were recruited in this study by the Sports Medicine Department at Peking University Third Hospital. Cartilages were harvested from the femoral condyles and tibial plateau during the total knee replacement surgery. The samples were then embedded in OCT compound for serial frozen sectioning. The sections were then incubated with DHE for 30 min at 37 °C, followed by three washes with PBS and mounted (Antifade Mounting Medium with DAPI). The photomicrographs were obtained using a confocal laser scanning microscope. The specimens was distinguished according to the severity of cartilage wear and synovitis in patients with OA. All procedures were approved by the ethics committee of Peking University Third Hospital and informed consent was obtained from each patient.

### Mouse Femoral Head Degradation Assay

Mouse femoral heads were harvested from 12‐week‐old BALB/c mice and cultured in DMEM medium for 24 h. Following the culture, 200 µM of H_2_O_2_ was added to stimulate cartilage degradation with or without 150 µg mL^−1^ of different MPs was added to the culture on days 0, 3, and 5. Culture medium was changed each time before H_2_O_2_ and MPs were added. A total of three treatments were performed. After a 7‐day incubation, femoral heads were collected and washed with PBS and fixed with 4% paraformaldehyde or rapidly frozen in OCT compound. For HE, Safranin O/fast green, toluidine blue, and immunohistochemical staining, mouse femoral heads were decalcified for 4 days before being dehydrated and embedded in paraffin. Then, 4 µm‐thick sections were cut and performed the above staining. For DHE staining, 8 µm‐thick cryosections were obtained and stained using the same procedure as described above.

### Mouse Models of Acute Osteoarthritis

To establish OA models, a single intra‐articular injection of 0.3 mg MIA in 25 µL of PBS was performed in the right knee joint of each mouse. One day after OA induction, the mice were randomly divided into five groups (*n* = 8). On days 1, 8, and 15, each group received an injection of either 25 µL of PBS (positive control) or 25 µL of PBS containing 25 µg of AKT‐MPs, MS‐AKT‐MPs, Mn‐AKT‐MPs, or Co‐AKT‐MPs, respectively. Mice (*n* = 8) without intra‐articular injection of MIA but with PBS administration were employed as a negative control. On day 23, the mice were euthanized, and the major organs and articular joints were collected for further analysis.

### In Vivo ROS Measurement

After MIA injection and different treatments for 7 days, changes in ROS levels in the inflamed knees in living mice were measured using chemiluminescent probe L‐012 (Wako Chemicals, Japan). Briefly, 100 µL of 500 µmol L^−1^ L‐012 solution was administered subcutaneously into the dorsal neck region of mice using an insulin syringe. After an incubation period of 30 min, the mice were anesthetized with isoflurane. The supine‐positioned luminescence images of affected joints were captured using an IVIS Spectrum system (Caliper, USA), and their intensities were quantified using Living Image software (version number, 4.3.1.16427).

### In Vivo Pro‐inflammatory Cytokine Secretion Analysis

The measurement of pro‐inflammatory cytokines in homogenized knee joints was performed using TNF‐α, IL‐1β, and MMP13 ELISA kits. Briefly, in 1 mL PBS, the right knee joints of mice after different treatments for 23 days were isolated and homogenized using the Tissue‐Tearor system. The homogenized solution was then centrifuged at 5000 g for 15 min at 4 °C. The pellet was discarded, and the supernatant was collected. Cytokine concentration in the supernatant was measured by TNF‐α, IL‐1β, and MMP13 ELISA kits following the manufacturer's instructions.

### Mouse Behavioral Tests

Testing for the sensitivity to the normal movement of each knee was performed according to the knee‐bend test previously described.^[^ ^40^ ^]^ Briefly, before the knee‐bend test, mice were allowed to adapt to the environment and gently restrained while at the same time allowing access to both hind limbs. The test consisted of the record of the number of squeaks and struggle reactions in response to 5 flexions and 5 extensions of the knee joint. Based on the following evaluation scale, the test score is determined: score 0 is given to joints that do not respond to any extension or flexion; for joints that struggle to reach their maximum flexion or extension, they are given a score of 0.5; struggles in moderate flexion/extension as well as vocalizations to maximal flexion/extension are given a score of 1; squeak reactions in response to moderate joint manipulations (flexions and extensions) are scored as 2. The total of the recorded reactions, with a maximum value of 20, represents the knee‐bend score, which indicates the animal's nociception.

### Histopathology

At the study endpoint, mouse stifles were collected and fixed in 4% paraformaldehyde for 2 days, followed by 4 weeks of decalcification in 0.5 M EDTA before paraffin embedding. As a standard procedure, fixed tissues were processed using graded alcohols, xylenes, and paraffin wax for 8 h. After embedding in paraffin wax, a series of sagittal sections with 4 µm thick were cut across the entire joint medial compartment. Staining was then performed using HE, Safranin O/Fast Green, and toluidine blue according to the manufacturer's instructions. Mouse knee sagittal sections stained with H&E were used to grade synovial membrane inflammation scores. Synovitis was assessed morphologically based on the following parameters: number of lining cell layers; proliferation of subsynovial tissue; infiltration of inflammatory cells. The synovitis score is graded from 0 to 4 according to severity. The measurement of the Mankin score was carried out as described previously.^[^ ^41^ ^]^ Briefly, a total of two sections within every six consecutive sections on each knee were stained with Safranin O/Fast Green, and a single score was assigned to each knee, representing the maximum score of its sections. An ImageJ analysis was conducted to quantify the safranin‐O‐positive area.

For immunohistochemical analysis, 4‐ µm sections were incubated with 0.2% Triton X‐100 for permeabilization, blocked with 2% BSA for 1 h, and then incubated overnight at 4 °C with primary antibodies for COL‐1, COL‐2, COL‐10. After washing with PBS, the sections were incubated with biotinylated anti‐rabbit IgG for chromagen development and counterstained with haematoxylin for visualization of cell nuclei. Micromaster II Microscope was used to image the samples. Histological scoring and quantitative staining analyses were all performed in a double‐blinded manner.

### Micro‐Computed Tomography

After 23 days of exposure to various treatments, affected knee joints were obtained, and the surrounding soft tissue, including skin and muscles, was removed and fixed in formalin for 2 days. The samples were then scanned using a micro‐CT (Inveon Scanners, Siemens, Germany) with 55 kV x‐ray potential, 145 µA current, and 314 ms integration time. 3D reconstruction and tibial subchondral bone analysis of scanned images were performed using Inveon Research Workplace. The histomorphometric analysis was performed in the coronal images of the tibial subchondral bone, including the ratio of bone volume to tissue volume (BV/TV), trabecular number (Tb. N), trabecular separation (Tb. Sp), trabecular thickness (Tb. Th), and trabecular pattern factor (Tb. Pf).

### Statistical Analysis

The data are expressed as mean ± standard deviation (SD), one‐way analysis of variance (ANOVA) or two‐way ANOVA with Tukey's posthoc test by using GraphPad Prism software version 8.0 (CA, USA). Statistical significance was set as follows: ^*^ *p* < 0.05, ^**^ *p* < 0.01, ^***^ *p* < 0.001, ^****^ *p* < 0.0001. Values of *p* < 0.05 were considered statistically significant, and ns denotes no significant difference.

## Conflict of Interest

The authors declare no conflict of interest.

## Supporting information



Supporting Information

## Data Availability

The data that support the findings of this study are available from the corresponding author upon reasonable request.

## References

[1] a) L. Sharma , N. Engl. J. Med. 2021, 384, 51; 33406330 10.1056/NEJMcp1903768

[2] N. K. Arden , T. A. Perry , R. R. Bannuru , O. Bruyere , C. Cooper , I. K. Haugen , M. C. Hochberg , T. E. McAlindon , A. Mobasheri , J. Y. Reginster , Nat. Rev. Rheumatol. 2021, 17, 59. 33116279 10.1038/s41584-020-00523-9

[3] a) S. Muthu , J. V. Korpershoek , E. J. Novais , G. F. Tawy , A. P. Hollander , I. Martin , Nat. Rev. Rheumatol. 2023, 19, 403; 37296196 10.1038/s41584-023-00979-5

[4] D. J. Hunter , S. Bierma‐Zeinstra , Lancet 2019, 393, 1745. 31034380 10.1016/S0140-6736(19)30417-9

[5] Q. Yao , X. Wu , C. Tao , W. Gong , M. Chen , M. Qu , Y. Zhong , T. He , S. Chen , G. Xiao , Signal Transduct. Target. Ther. 2023, 8, 56. 36737426 10.1038/s41392-023-01330-w PMC9898571

[6] a) W. H. Robinson , C. M. Lepus , Q. Wang , H. Raghu , R. Mao , T. M. Lindstrom , J. Sokolove , Nat. Rev. Rheumatol. 2016, 12, 580; 27539668 10.1038/nrrheum.2016.136 PMC5500215

[7] R. F. Loeser , J. A. Collins , B. O. Diekman , Nat. Rev. Rheumatol. 2016, 12, 412. 27192932 10.1038/nrrheum.2016.65 PMC4938009

[8] T. Gui , L. Luo , B. Chhay , L. Zhong , Y. Wei , L. Yao , W. Yu , J. Li , C. L. Nelson , A. Tsourkas , L. Qin , Z. Cheng , Biomaterials 2022, 283, 121437. 35247635 10.1016/j.biomaterials.2022.121437 PMC8977249

[9] H. Lu , J. Wei , K. Liu , Z. Li , T. Xu , D. Yang , Q. Gao , H. Xiang , G. Li , Y. Chen , ACS Nano 2023, 17, 6131. 36920036 10.1021/acsnano.3c01789

[10] a) C. Shu , C. Qin , L. Chen , Y. Wang , Z. Shi , J. Yu , J. Huang , C. Zhao , Z. Huan , C. Wu , M. Zhu , Y. Zhu , Adv. Sci. (Weinh) 2023, 10, 2206875; 36828785 10.1002/advs.202206875 PMC10161093

[11] M. Arra , G. Swarnkar , K. Ke , J. E. Otero , J. Ying , X. Duan , T. Maruyama , M. F. Rai , R. J. O'Keefe , G. Mbalaviele , J. Shen , Y. Abu‐Amer , Nat. Commun. 2020, 11, 3427. 32647171 10.1038/s41467-020-17242-0 PMC7347613

[12] a) R. H. Deng , M. Z. Zou , D. Zheng , S. Y. Peng , W. Liu , X. F. Bai , H. S. Chen , Y. Sun , P. H. Zhou , X. Z. Zhang , ACS Nano 2019, 13, 8618; 31246413 10.1021/acsnano.9b02993

[13] S. Zhang , L. Wang , Y. Kang , J. Wu , Z. Zhang , Acta Biomater. 2023, 162, 1. 36967052 10.1016/j.actbio.2023.03.030

[14] X. Gao , Y. Ma , G. Zhang , F. Tang , J. Zhang , J. Cao , C. Liu , Int. J. Pharm. 2020, 590, 119947. 33031875 10.1016/j.ijpharm.2020.119947

[15] a) P. H. Canter , B. Wider , E. Ernst , Rheumatology (Oxford) 2007, 46, 1223; 17522095 10.1093/rheumatology/kem116

[16] a) P. Yu , Y. Li , H. Sun , H. Zhang , H. Kang , P. Wang , Q. Xin , C. Ding , J. Xie , J. Li , Adv. Mater. 2023, 35, 2303299; 10.1002/adma.202303299 37459592

[17] A. G. Bajpayee , A. J. Grodzinsky , Nat. Rev. Rheumatol. 2017, 13, 183. 28202920 10.1038/nrrheum.2016.210

[18] S. K. Bedingfield , J. M. Colazo , M. Di Francesco , F. Yu , D. D. Liu , V. Di Francesco , L. E. Himmel , M. K. Gupta , H. Cho , K. A. Hasty , P. Decuzzi , C. L. Duvall , ACS Nano 2021, 15, 14475. 34409835 10.1021/acsnano.1c04005 PMC9074946

[19] a) S. K. Bedingfield , J. M. Colazo , F. Yu , D. D. Liu , M. A. Jackson , L. E. Himmel , H. Cho , L. J. Crofford , K. A. Hasty , C. L. Duvall , Nat. Biomed. Eng. 2021, 5, 1069; 34413494 10.1038/s41551-021-00780-3 PMC8497446

[20] M. C. Bruno , M. C. Cristiano , C. Celia , N. d'Avanzo , A. Mancuso , D. Paolino , J. Wolfram , M. Fresta , ACS Nano 2022, 16, 19665. 36512378 10.1021/acsnano.2c06393

[21] J. Paik , S. T. Duggan , S. J. Keam , Drugs 2019, 79, 455. 30847805 10.1007/s40265-019-01083-3 PMC6437125

[22] a) Y. Huang , J. Ren , X. Qu , Chem. Rev. 2019, 119, 4357; 30801188 10.1021/acs.chemrev.8b00672

[23] a) C. Peng , R. Pang , J. Li , E. Wang , Adv. Mater. 2023, 36, 2211724; 10.1002/adma.202211724 36773312

[24] F. Cao , L. Zhang , Y. You , L. Zheng , J. Ren , X. Qu , Angew. Chem., Int. Ed. Engl. 2020, 59, 5108. 31960567 10.1002/anie.201912182

[25] a) J. Zhang , Y. Fang , W. Zhao , R. Han , J. Wen , S. F. Liu , Adv. Mater. 2021, 33, 2103770; 10.1002/adma.202103770 34554617

[26] Q. Pang , J. Meng , S. Gupta , X. Hong , C. Y. Kwok , J. Zhao , Y. Jin , L. Xu , O. Karahan , Z. Wang , S. Toll , L. Mai , L. F. Nazar , M. Balasubramanian , B. Narayanan , D. R. Sadoway , Nature 2022, 608, 704. 36002488 10.1038/s41586-022-04983-9

[27] S. Kaushik , D. Wu , Z. Zhang , X. Xiao , C. Zhen , W. Wang , N. Y. Huang , M. Gu , Q. Xu , Adv. Mater. 2024, 36, 2401163. 10.1002/adma.202401163 38639567

[28] a) J. Zhang , Y. Fang , W. Zhao , R. Han , J. Wen , S. F. Liu , Adv. Mater. 2021, 33, 2103770; 10.1002/adma.202103770 34554617

[29] C. Deng , Q. Zhou , M. Zhang , T. Li , H. Chen , C. Xu , Q. Feng , X. Wang , F. Yin , Y. Cheng , C. Wu , Adv. Sci. (Weinh) 2022, 9, 2105727. 35182053 10.1002/advs.202105727 PMC9036007

[30] M. Xiao , L. Zhang , B. Luo , M. Lyu , Z. Wang , H. Huang , S. Wang , A. Du , L. Wang , Angew. Chem., Int. Ed. Engl. 2020, 59, 7230. 32067299 10.1002/anie.202001148

[31] Y. Li , H. Shao , Z. Lin , J. Lu , L. Liu , B. Duployer , P. O. A. Persson , P. Eklund , L. Hultman , M. Li , K. Chen , X. H. Zha , S. Du , P. Rozier , Z. Chai , E. Raymundo‐Pinero , P. L. Taberna , P. Simon , Q. Huang , Nat. Mater. 2020, 19, 894. 32284597 10.1038/s41563-020-0657-0

[32] T. Ebata , M. A. Terkawi , K. Kitahara , S. Yokota , J. Shiota , Y. Nishida , G. Matsumae , H. Alhasan , M. Hamasaki , K. Hontani , T. Shimizu , D. Takahashi , T. Endo , T. Onodera , K. Kadoya , N. Iwasaki , Arthritis Rheumatol. 2023, 75, 1358. 36924130 10.1002/art.42505

[33] H. Sies , Redox Biol. 2017, 11, 613. 28110218 10.1016/j.redox.2016.12.035 PMC5256672

[34] C. Shi , Q. Zhang , Y. Yao , F. Zeng , C. Du , S. Nijiati , X. Wen , X. Zhang , H. Yang , H. Chen , Z. Guo , X. Zhang , J. Gao , W. Guo , X. Chen , Z. Zhou , Nat. Nanotechnol. 2023, 18, 86. 36536041 10.1038/s41565-022-01261-7

[35] W. L. Wan , Y. J. Lin , P. C. Shih , Y. R. Bow , Q. Cui , Y. Chang , W. T. Chia , H. W. Sung , Angew. Chem., Int. Ed. Engl. 2018, 57, 9875. 29923670 10.1002/anie.201806159

[36] J. Martel‐Pelletier , A. J. Barr , F. M. Cicuttini , P. G. Conaghan , C. Cooper , M. B. Goldring , S. R. Goldring , G. Jones , A. J. Teichtahl , J. P. Pelletier , Nat. Rev. Dis. Primers 2016, 2, 16072. 27734845 10.1038/nrdp.2016.72

[37] H. Zhang , C. Lin , C. Zeng , Z. Wang , H. Wang , J. Lu , X. Liu , Y. Shao , C. Zhao , J. Pan , S. Xu , Y. Zhang , D. Xie , D. Cai , X. Bai , Ann. Rheum. Dis. 2018, 77, 1524. 29991473 10.1136/annrheumdis-2018-213450

[38] L. Leyssens , B. Vinck , C. Van Der Straeten , F. Wuyts , L. Maes , Toxicology 2017, 387, 43. 28572025 10.1016/j.tox.2017.05.015

[39] N. A. Hodges , E. M. Sussman , J. P. Stegemann , Biomaterials 2021, 278, 121127. 34564034 10.1016/j.biomaterials.2021.121127

[40] J. Ferreira‐Gomes , S. Adaes , J. M. Castro‐Lopes , J. Pain 2008, 9, 945. 18650131 10.1016/j.jpain.2008.05.012

[41] T. Aigner , J. L. Cook , N. Gerwin , S. S. Glasson , S. Laverty , C. B. Little , W. McIlwraith , V. B. Kraus , Osteoarthritis Cartilage 2010, 18, S2. 20864020 10.1016/j.joca.2010.07.013

