# Supplementary material for: Molten‐Salt‐Mediated Synthesis of Atomic Manganese/Cobalt Catalysts on Bioceramic Microparticles for Catalytic Anti‐Osteoarthritis Treatments
Source: Adv Sci (Weinh). 2025 Jul 21;12(31):e05500. doi: 10.1002/advs.202505500 (PMC12376603; doi:10.1002/advs.202505500)
Supplement: Supplementary file 1 — Supporting Information [file ADVS-12-e05500-s001.docx]

Supporting Information

Molten-Salt-Mediated Synthesis of Atomic Manganese/cobalt Catalysts on Bioceramic Microparticles for Catalytic Anti-Osteoarthritis Treatments

*Ronghui Deng^1^**^,2,3†^, Zining Zhang^1,2†^, Aijun Wu^4†^, Chaoqin Shu^4^, Shitang Song^1,2^, Fuzhen Yuan^1,2^, Zijie Xu^1,2^, Meng Yang^1,2^,Jing Ye^1,2^, Yifan Song^1,2^, Yufang Zhu^4,5^*, Jia-Kuo Yu^1,2,3^**

^1^Sports Medicine Department, Beijing Key Laboratory of Sports Injuries, Peking University Third Hospital, Beijing 100191, P. R. China

^2^Peking University Institute of Sports Medicine, Beijing 100191, P. R. China

^3^Orthopaedic and Sports Medicine Center, Beijing Tsinghua Changgung Hospital, Tsinghua University, Beijing 102218, P. R. China

^4^State Key Laboratory of High Performance Ceramics and Superfine Microstructure, Shanghai Institute of Ceramics, Chinese Academy of Sciences, Shanghai 200050, P. R. China

^5^Center of Materials Science and Optoelectronics Engineering, University of Chinese Academy of Sciences, Beijing 100049, P. R. China

^†^The authors contributed equally to this work.

*Corresponding authors:

Yufang Zhu, Ph.D., Professor

Shanghai Institute of Ceramics, Chinese Academy of Sciences, China

Email: zhuyufang@mail.sic.ac.cn

Jia-Kuo Yu, Ph.D., Professor

Peking University Third Hospital, China

Email: yujiakuo@126.com

**Supplementary Figures 1 to 18**

**
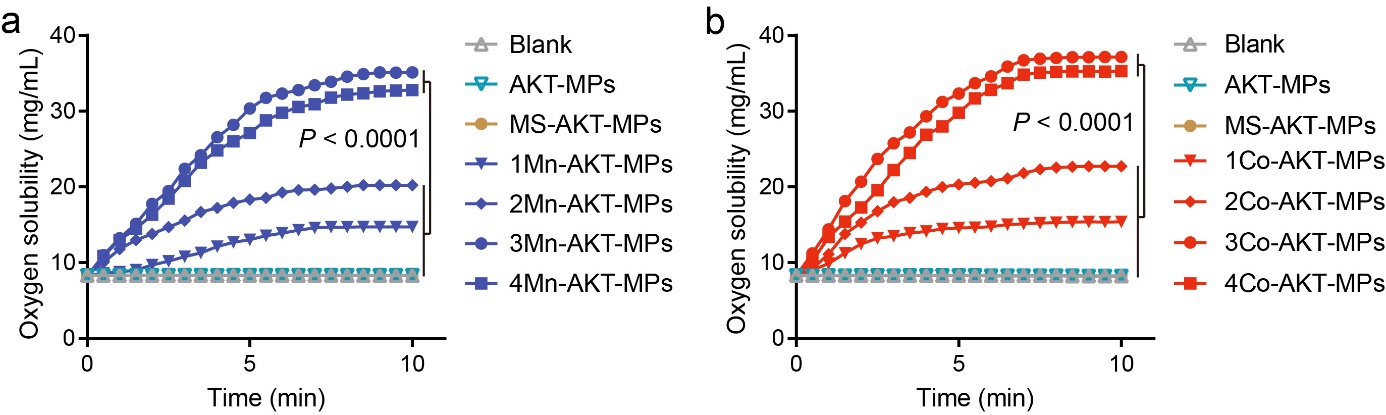
**

**Figure S1.** Production of O_2_ by Mn-AKT-MPs or Co-AKT-MPs with different additions of Mn or Co content prepared at 350 °C to scavenge H_2_O_2_. *P* values are shown in the graphs. Statistical significance was analyzed by two-way ANOVA with Tukey’s multiple comparisons test.

**
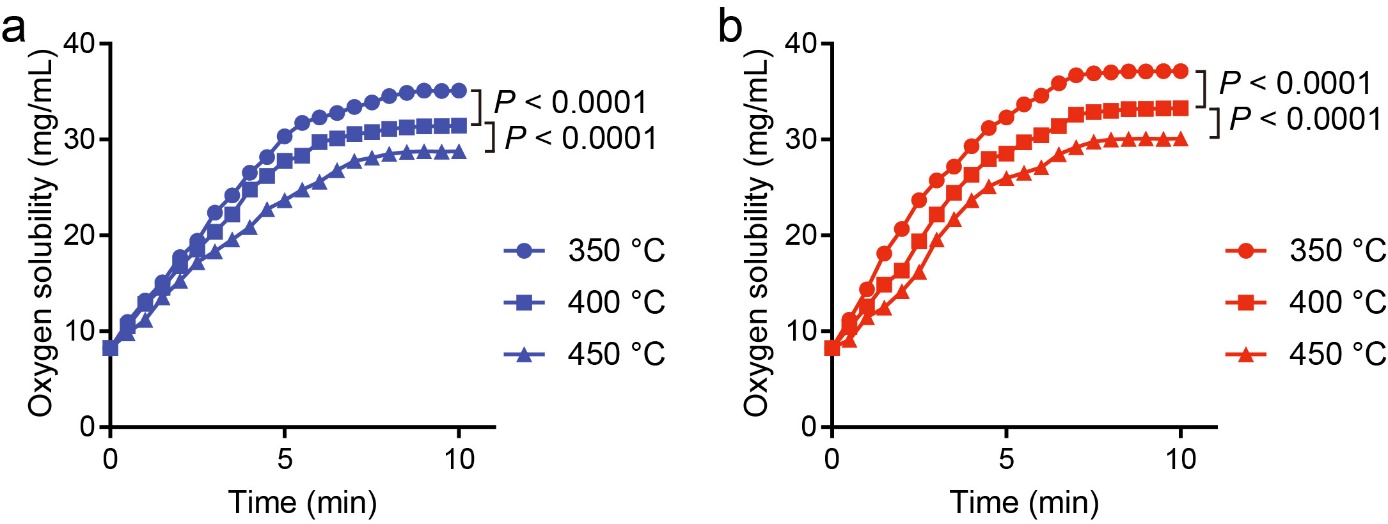
**

**Figure S2.** Production of O_2_ by the 3Mn-AKT-MPs or 3Co-AKT-MPs prepared at various temperatures to scavenge H_2_O_2_. *P* values are shown in the graphs. Statistical significance was analyzed by two-way ANOVA with Tukey’s multiple comparisons test.


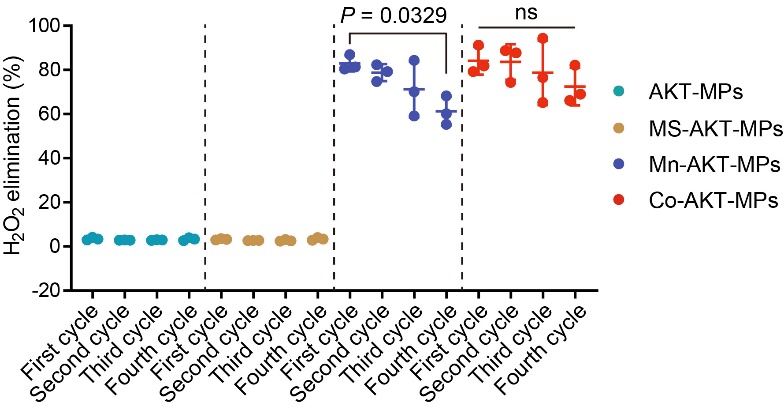


**Figure S3**. H_2_O_2_ scavenging activity of Mn-AKT-MPs or Co-AKT-MPs over four consecutive cycles. (n = 3).

**
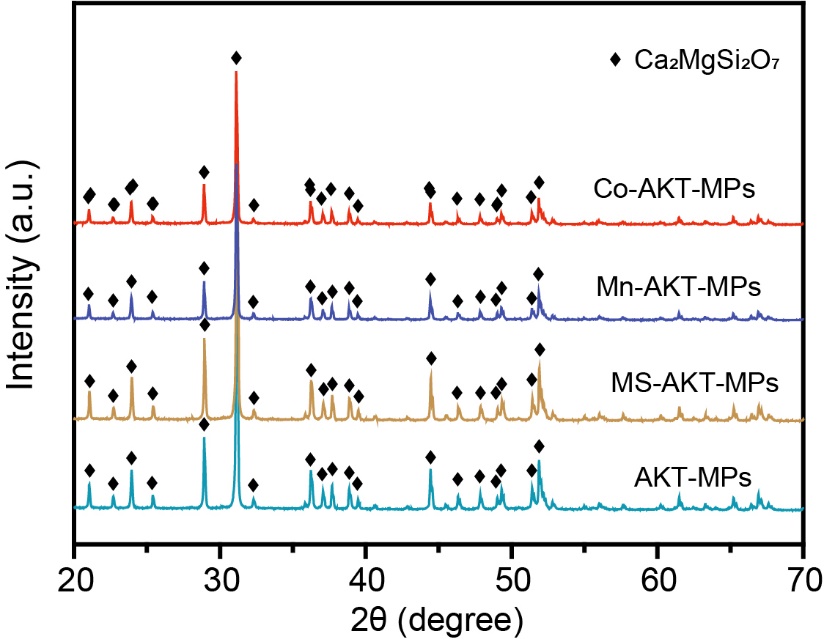
**

**Figure S4.** X-ray diffraction (XRD) analysis for phase composition of AKT-MPs, MS-AKT-MPs, Mn-AKT-MPs, and Co-AKT-MPs. The temperature of molten-salt treatment was at 350 °C.


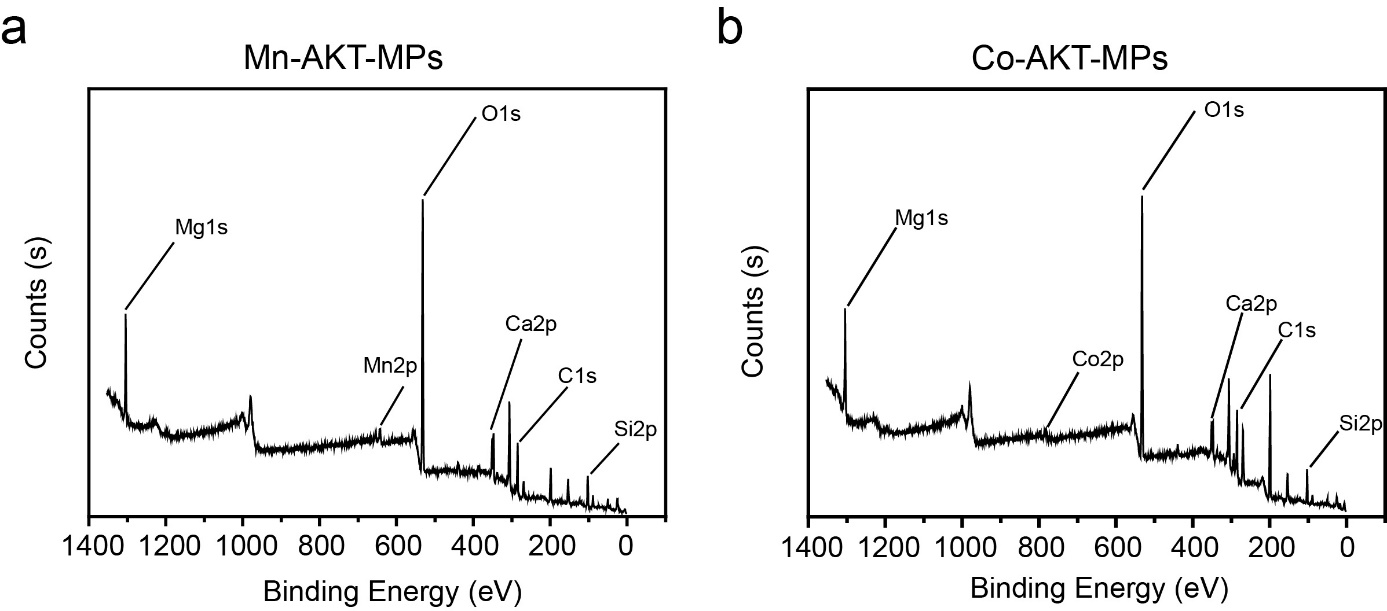


**Figure S5.** XPS spectra for (a) Mn-AKT-MPs and (b) Co-AKT-MPs.


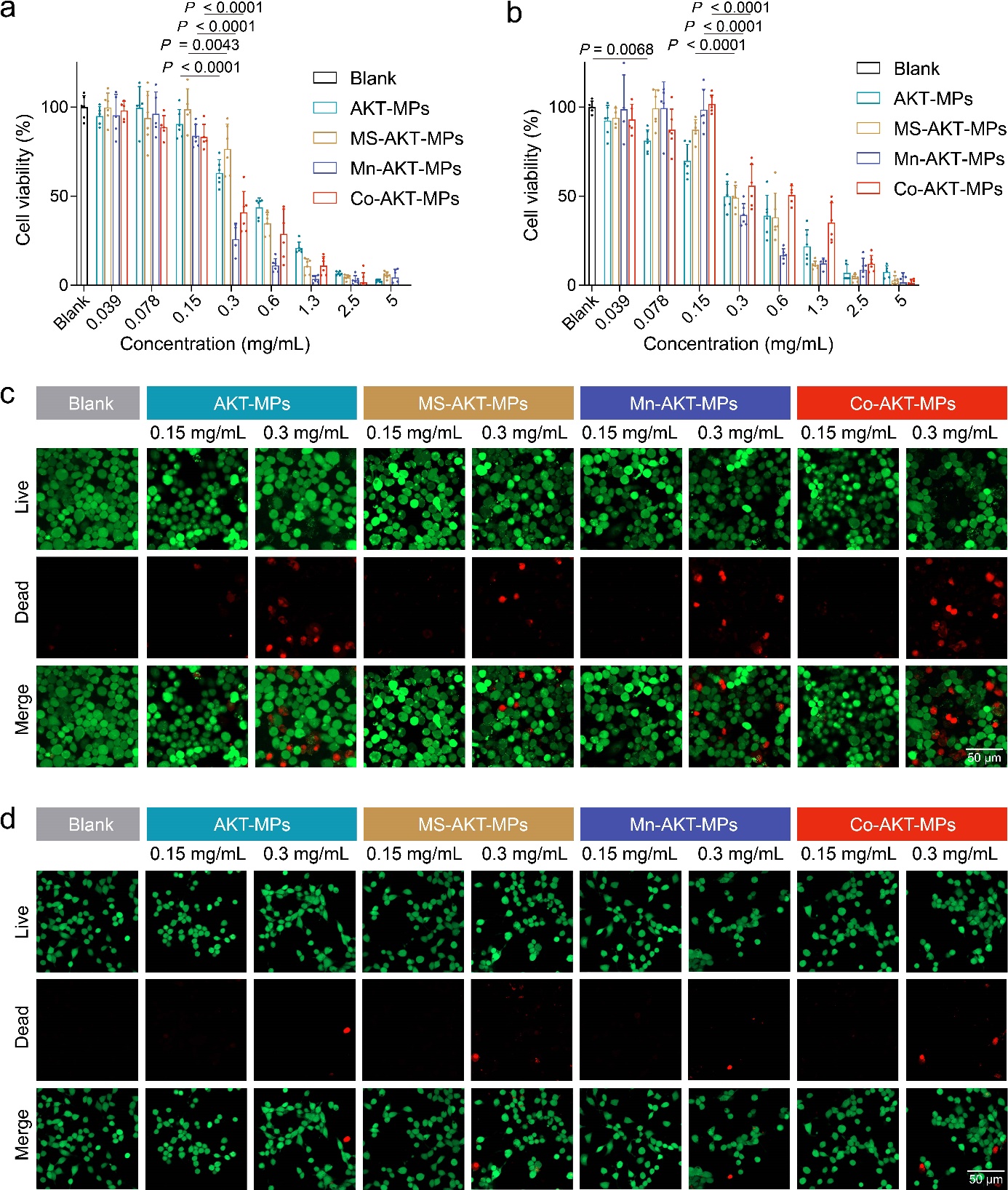


**Figure S6.** a-b) Concentration-dependent cell viability of RAW 264.7 (a) or ATDC5 (b) cells treated with AKT-MPs, MS-AKT-MPs, Mn-AKT-MPs, or Co-AKT-MPs for 24 h, determined by CCK-8 assay. c-d) Calcein-AM/PI staining of RAW 264.7 (c) or ATDC5 (d) cells after 24 h of incubation with 0.15 mg/mL or 0.3 mg/mL indicated formulations. Green, live; Red, Dead. Data are presented as the mean ± SD. *P* values are shown in the graphs. Statistical significance was analyzed by two-way ANOVA with Tukey’s multiple comparisons test.


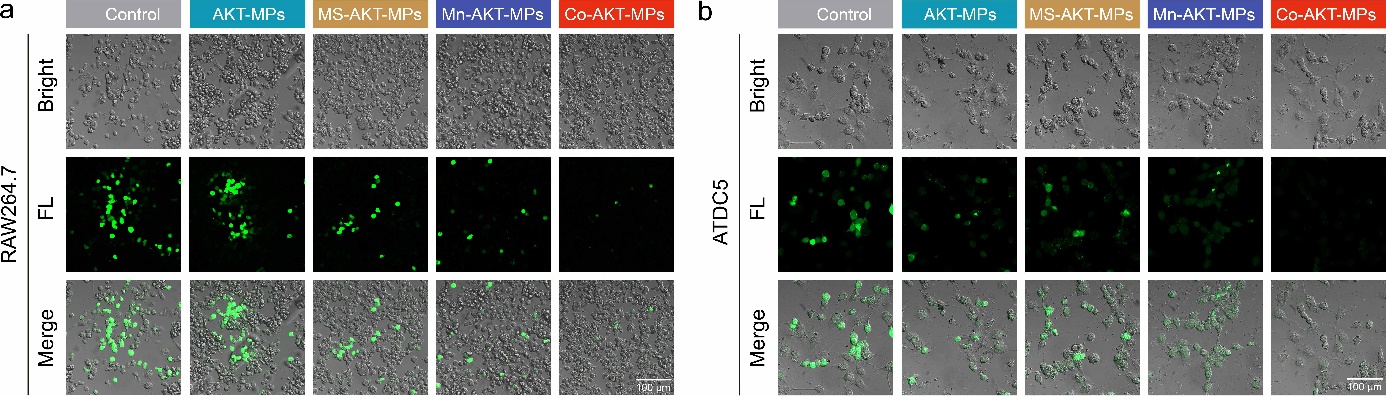


**Figure S7.** a-b) Representative confocal microscopy images of cellular ROS in H_2_O_2_-stimulated RAW 264.7 (a) and ATDC5 (b) cells after various treatments for 12 h. A ROS probe 2′,7′-dichlorofluorescein diacetate (DCFH-DA) was employed for cellular ROS detection, which could form 2′,7′-dichlorofluorescein (DCF) with green fluorescence emission when under ROS attack.


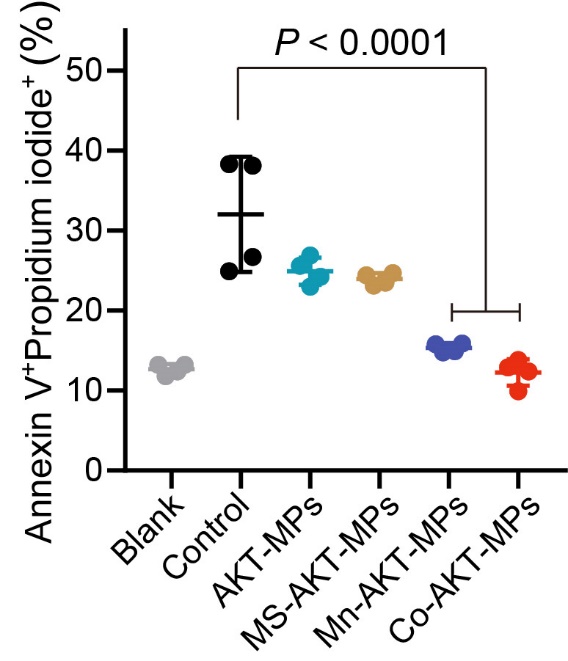


**Figure S8.** Quantitative analysis of Annexin V-FITC^+^/PI^+^ RAW 264.7 cells based on Annexin V-FITC/PI apoptosis staining (n = 4). Data are presented as the mean ± SD. *P* values are shown in the graphs. Statistical significance was analyzed by one-way ANOVA with Tukey’s multiple comparisons test.


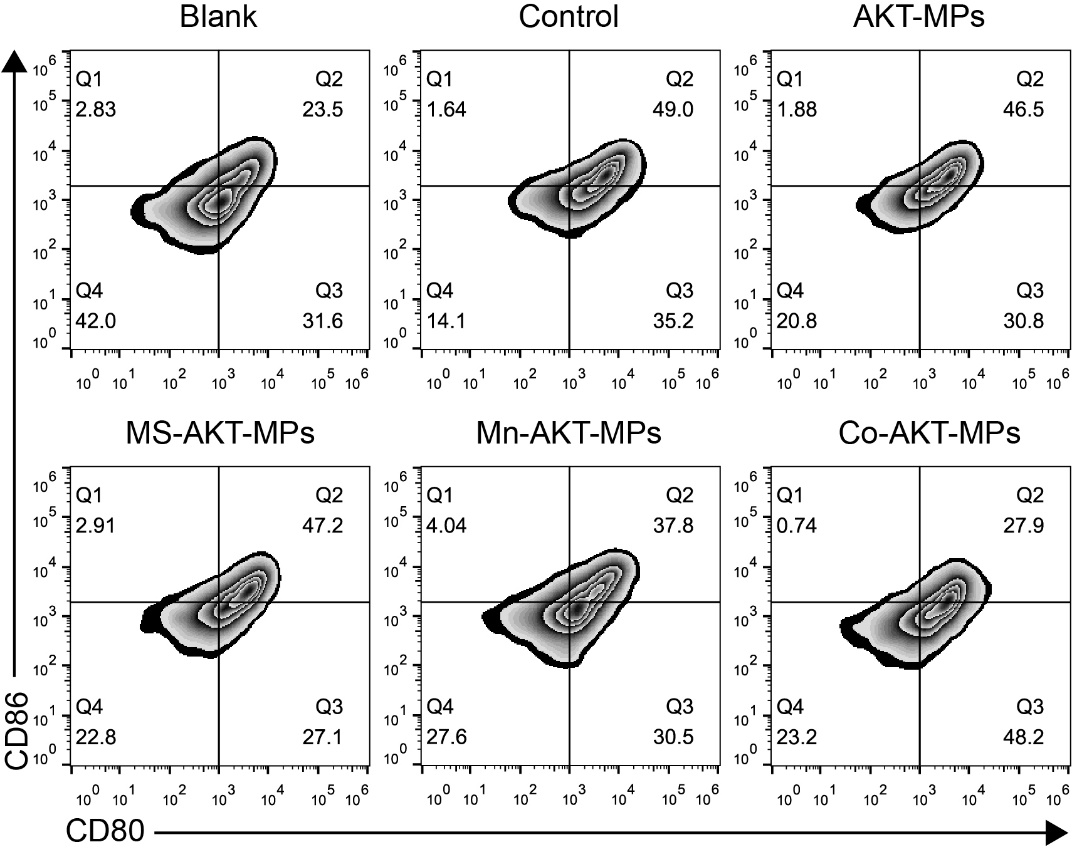


**Figure S9.** Flow cytometric analysis showing expression of CD80^+^CD86^+^ gating on CD11b^+^ H_2_O_2_-stimulated RAW 264.7 cells at 12 h time point following different treatments (n = 4).

**
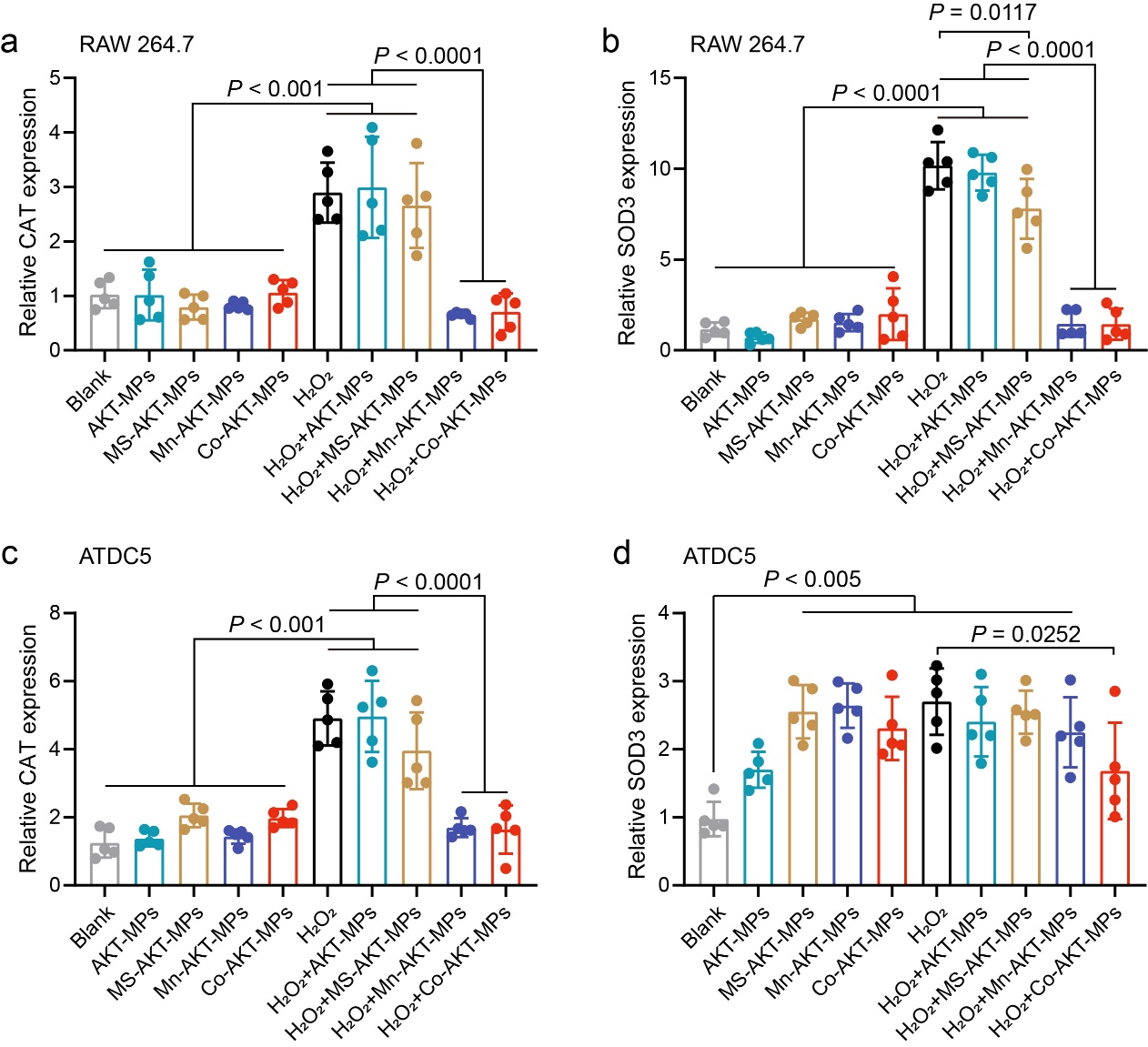
**

**Figure S10**. a-d) The relative gene expression of CAT and SOD3 was measured by quantitative real-time polymerase chain reaction (RT-PCR) in the blank, AKT-MPs-, MS-AKT-MPs-, Mn-AKT-MPs-, and Co-AKT-MPs-treated H_2_O_2_-stimulated or non-stimulated RAW 264.7 and ATDC5 cells (n = 5). *P* values are shown in the graphs. Statistical significance was analyzed by one-way ANOVA with Tukey’s multiple comparisons test.


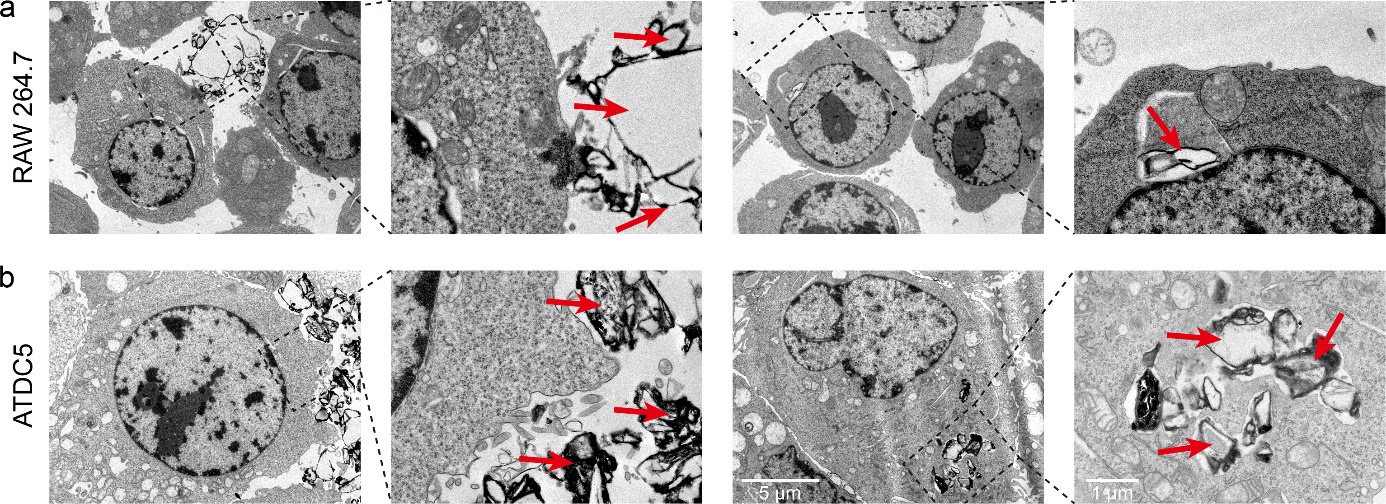


**Figure S11.** a-b) TEM images of RAW 264.7 (a) and ATDC5 (b) cells after incubated with Co-AKT-MPs for 12 h.

**
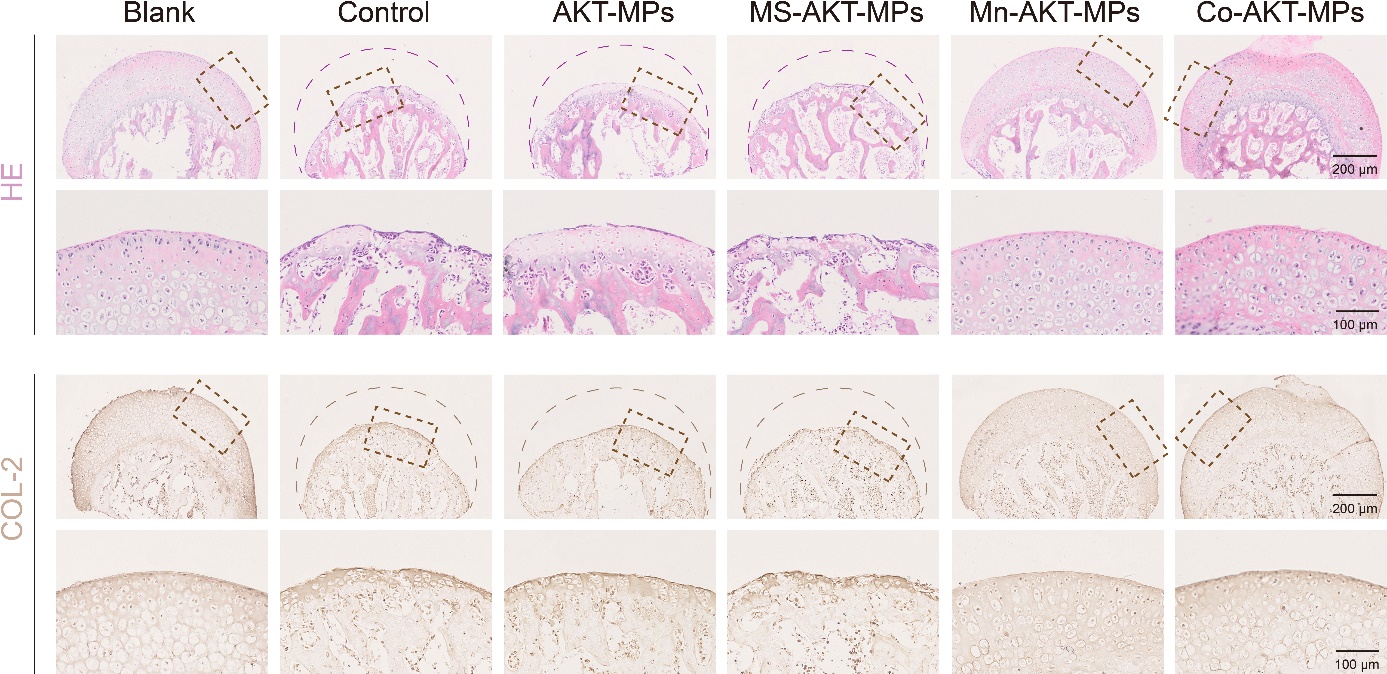
**

**Figure S12.** HE and immunohistochemical staining of COL-2 of mouse femoral heads under the indicated treatment conditions and continuous incubation with 200 μM H_2_O_2_ for 7 days. Bottom: Magnified images of the dashed boxed areas.

**
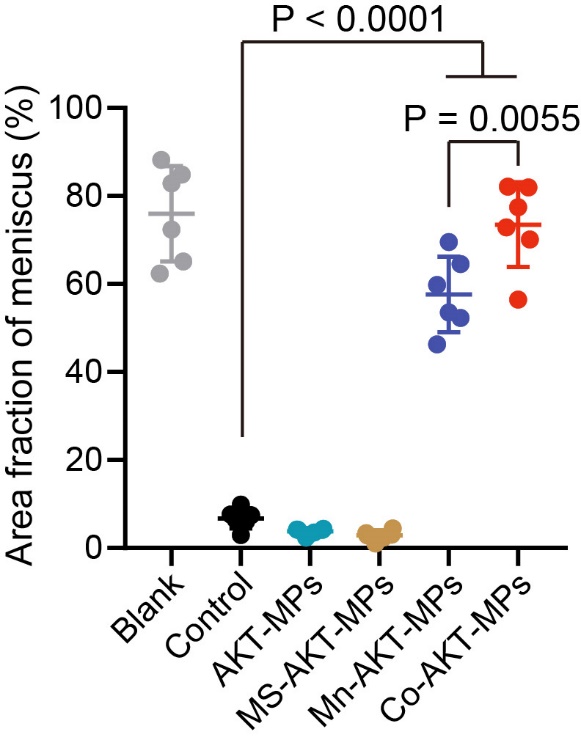
**

**Figure S13.** Corresponding quantitative analysis of safranin O-positive area in meniscus among the different groups (n = 6). Data are presented as the mean ± SD. *P* values are shown in the graphs. Statistical significance was analyzed by one-way ANOVA with Tukey’s multiple comparisons test.

**
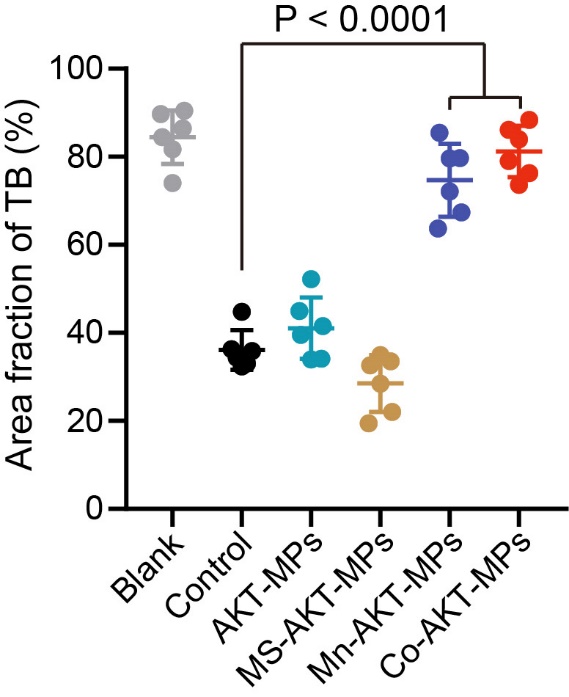
**

**Figure S14.** Corresponding quantitative analysis of toluidine blue-positive area in cartilage among the different groups (n = 6). Data are presented as the mean ± SD. *P* values are shown in the graphs. Statistical significance was analyzed by one-way ANOVA with Tukey’s multiple comparisons test.

**
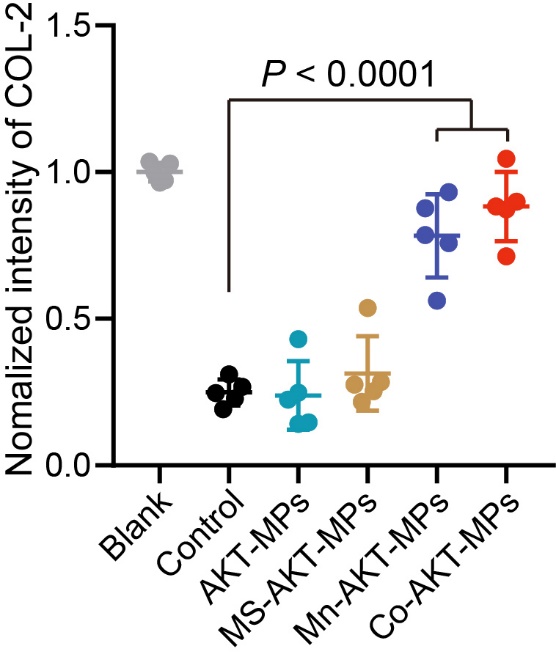
**

**Figure S15.** Corresponding quantitative analysis of COL-2 intensity in cartilage based on immunohistochemical staining among the different groups (n = 6). Data are presented as the mean ± SD. *P* values are shown in the graphs. Statistical significance was analyzed by one-way ANOVA with Tukey’s multiple comparisons test.

**
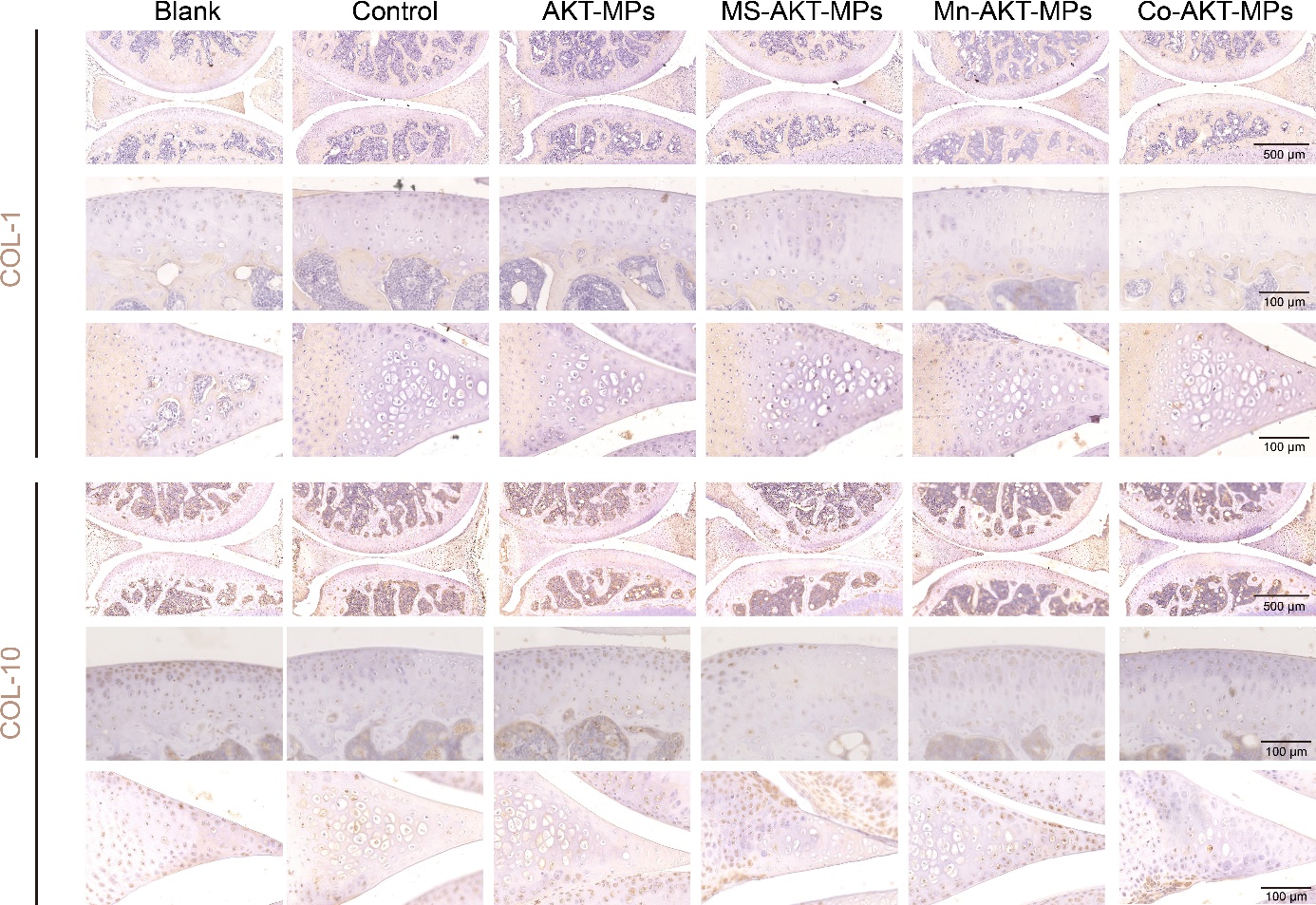
**

**Figure S16**. Immunohistochemical staining of COL-1 (top) and COL-10 (bottom) in cartilage and meniscus collected from various treated knee joins.


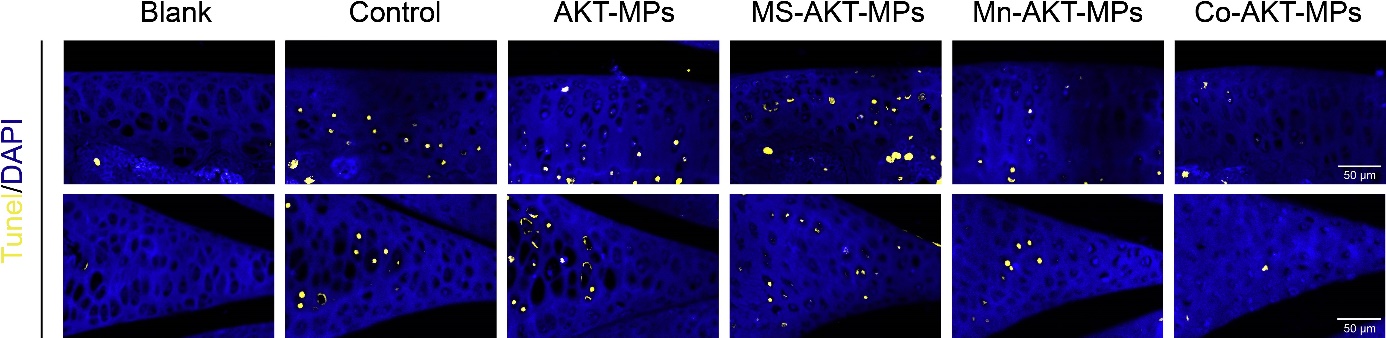


**Figure S17.** Tunel staining in cartilage and meniscus collected from the knee joins treated in various groups.


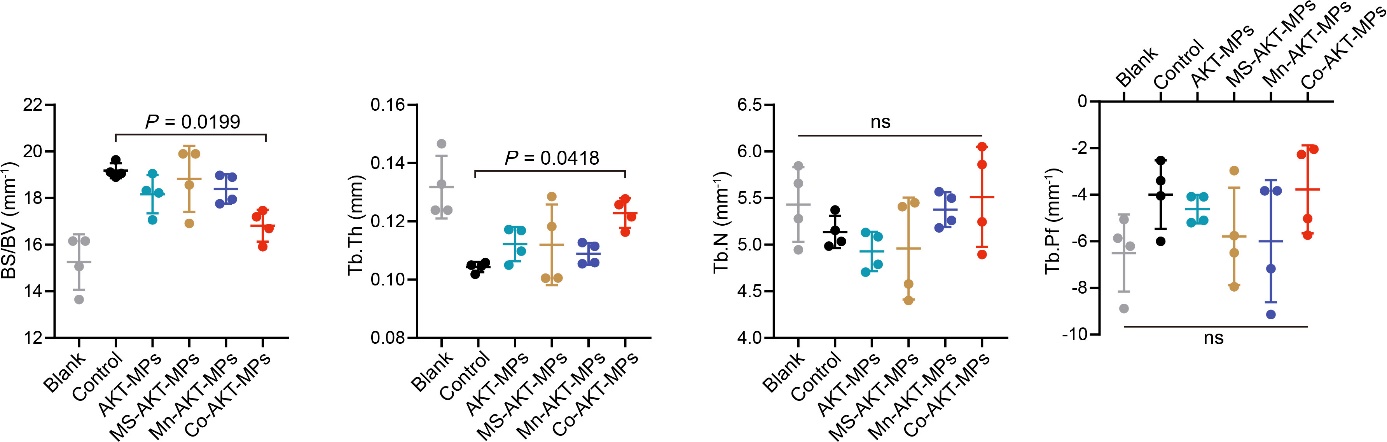


**Figure S18.** The parameters of subchondral bone micro-architecture were quantified (n = 4). BS/BV, bone surface/bone volume ratio; Tb.Th, trabecular thickness; Tb.N, trabecular number, Tb.Pf, trabecular pattern factor. Data are presented as the mean ± SD. *P* values are shown in the graphs; ns, no significance. Statistical significance was analyzed by one-way ANOVA with Tukey’s multiple comparisons test.


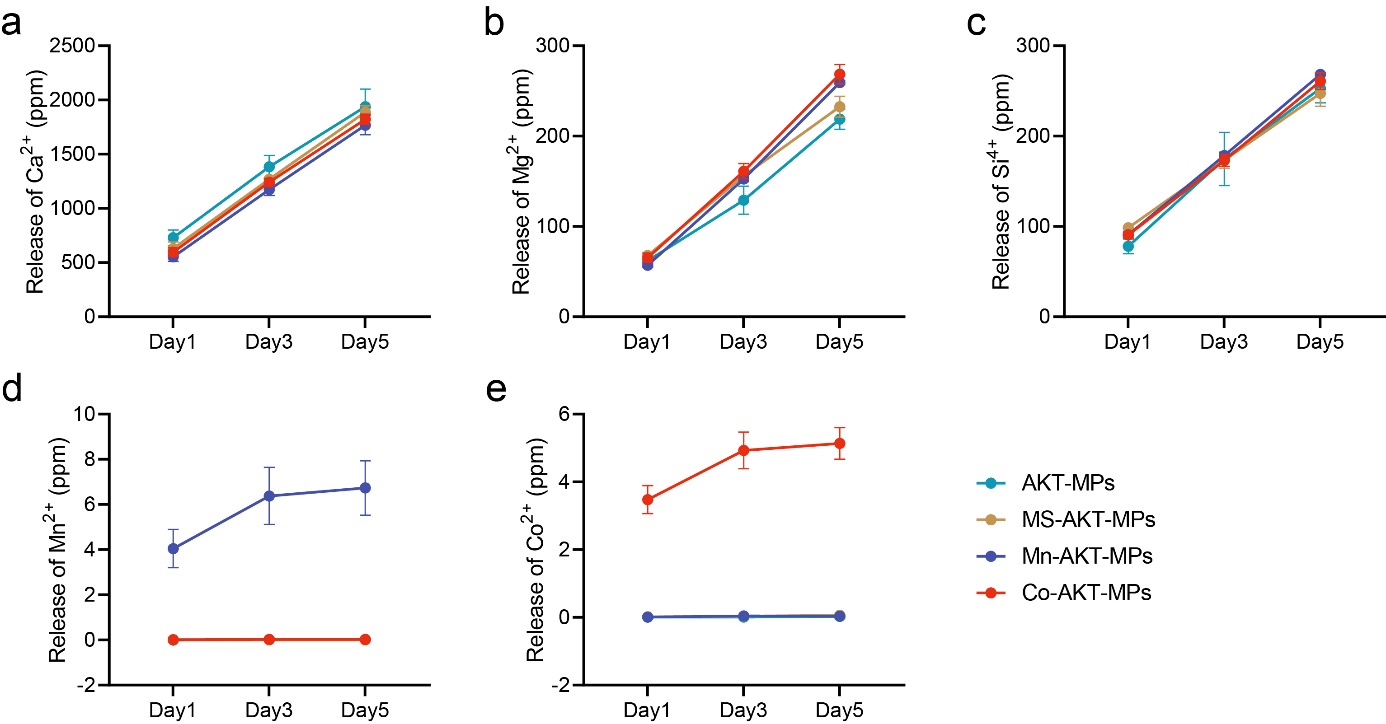


**Figure S19.** a-e) Cumulative release profiles of Ca^2+^, Mg^2+^, Si^4+^, Mn^2+^, and Co^2+^ ions from AKT-MPs, MS-AKT-MPs, Mn-AKT-MPs, and Co-AKT-MPs in Tris-HCl buffer solution at 1, 3, and 5 days, respectively (n = 3).


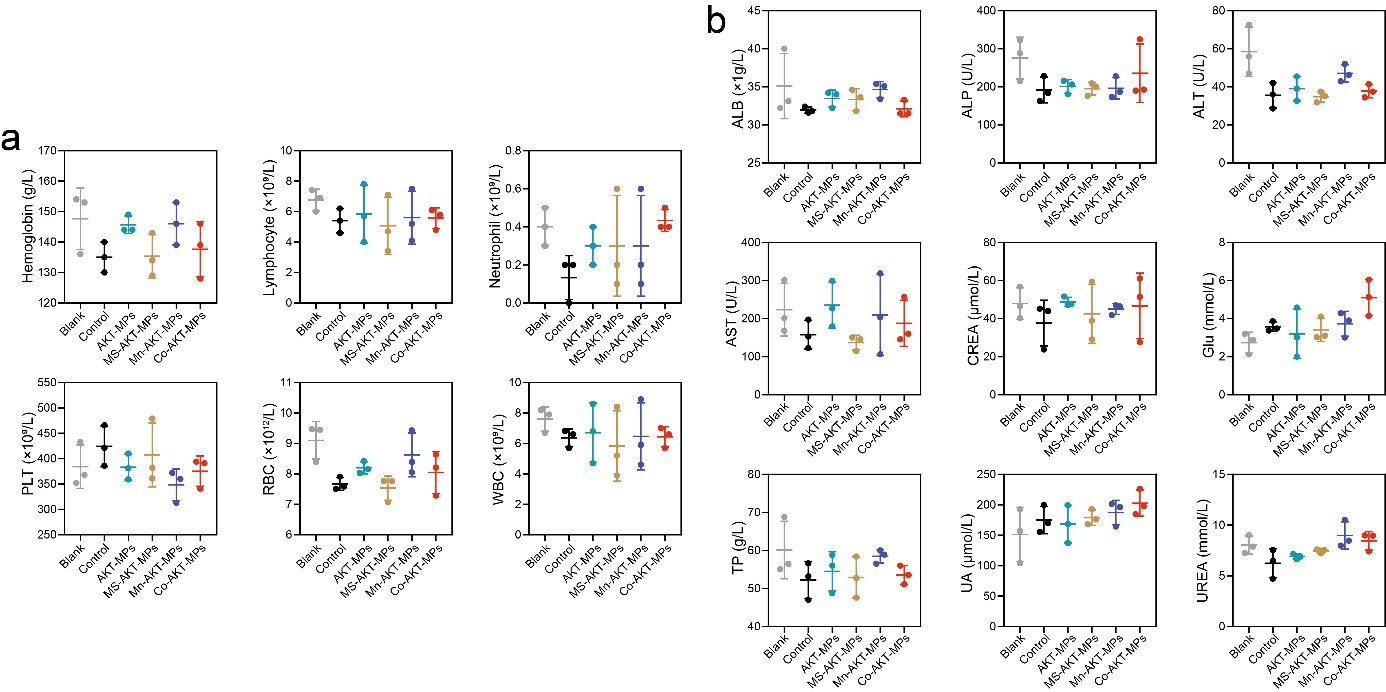


**Figure S20.** a) Biochemical parameters of the serum from the mice at the end of the experiment in the MIA induced OA model was performed to evaluate liver and renal function in different groups (n = 3). b) Hematological parameters of the mice at the end of the experiment in the MIA induced OA model (n = 3). There was no significant difference among groups for all measured parameters. Data are presented as the mean ± SD. Statistical significance was analyzed by one-way ANOVA with Tukey’s multiple comparisons test.

**
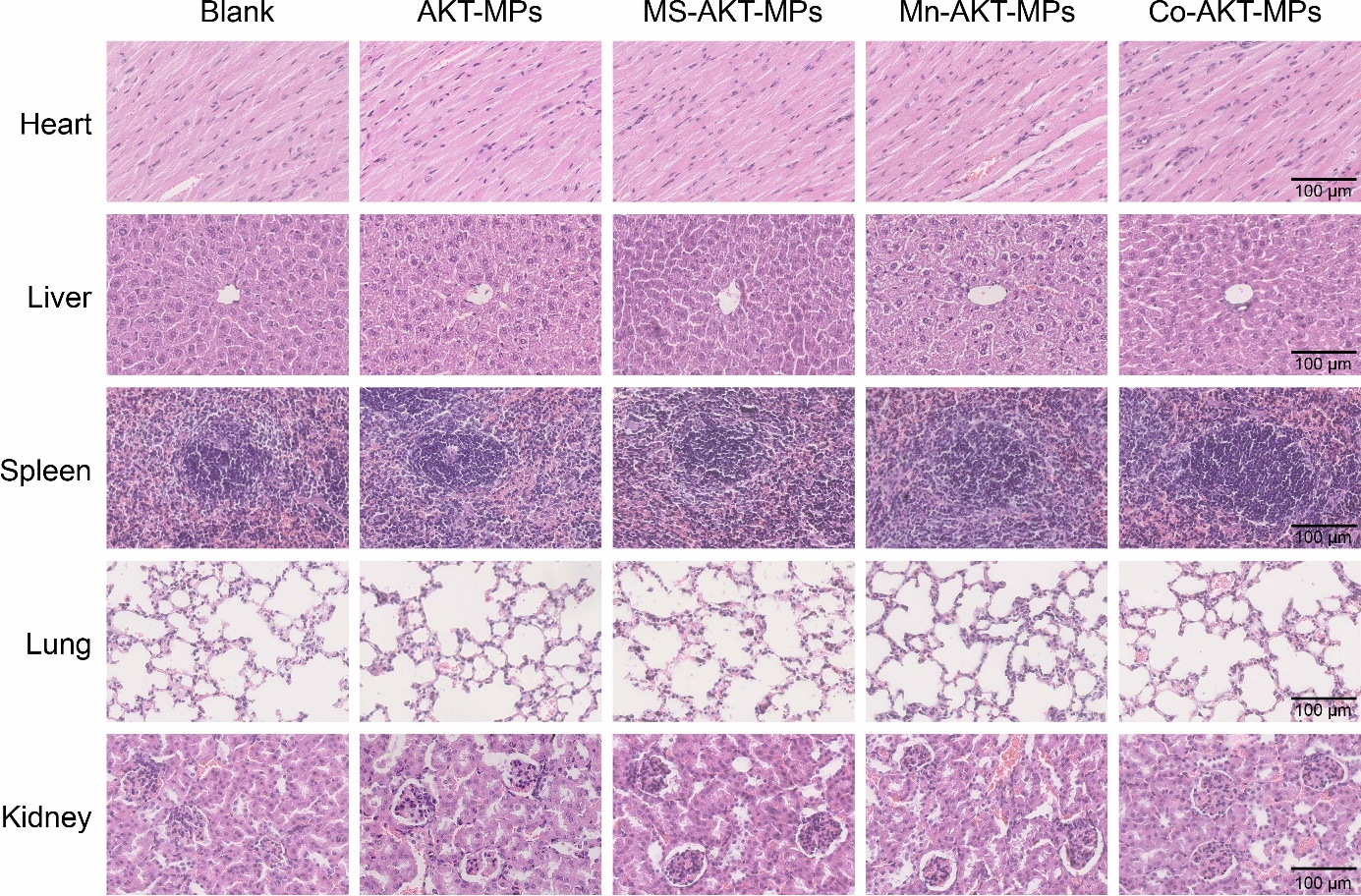
**

**Figure S21.** Representative HE staining of major organs (heart, liver, spleen, lung, and kidney) at the end of the experiment in the MIA induced OA model.


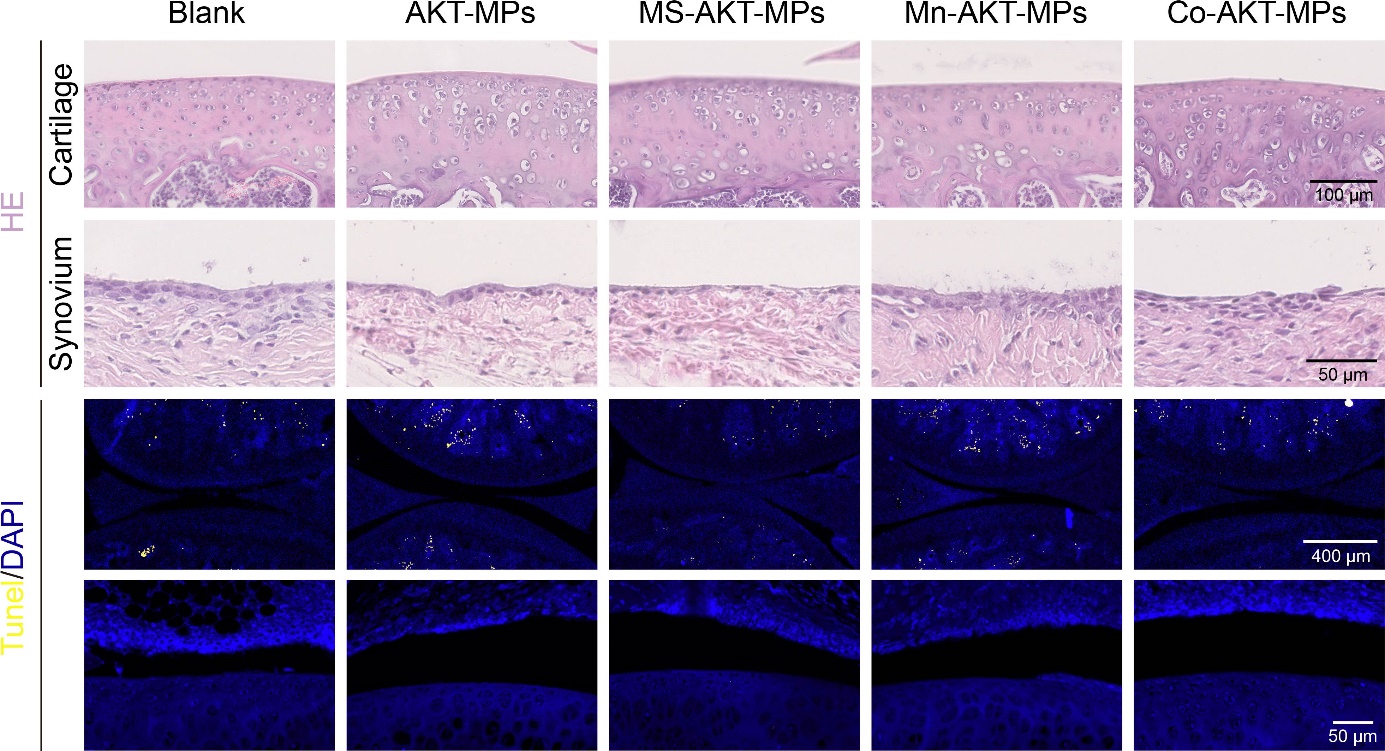


**Figure S22.** a) Representative HE and tunel staining of cartilage and synovium tissues from the mice without the injection of MIA and treated with indicated groups.

**Supplementary Tables 1 to 2**

**Table S1.** **Amounts of salt, AKT microparticles, and the precursor of Mn or Co in the synthesis process**

| **Name** | **AKT-MPs (g)** | **KCl (g)** | **LiCl (g)** | **MnCl_2_ (mg)** | **CoCl_2_·6H_2_O (mg)** |
| --- | --- | --- | --- | --- | --- |
| MS-AKT-MPs | 3.0 | 3.3 | 2.7 | 0 | 0 |
| 1Mn-AKT-MPs | 3.0 | 3.3 | 2.7 | 7.5 | 0 |
| 2Mn-AKT-MPs | 3.0 | 3.3 | 2.7 | 15.0 | 0 |
| 3Mn-AKT-MPs | 3.0 | 3.3 | 2.7 | 30.0 | 0 |
| 4Mn-AKT-MPs | 3.0 | 3.3 | 2.7 | 60.0 | 0 |
| 1Co-AKT-MPs | 3.0 | 3.3 | 2.7 | 0 | 14.0 |
| 2Co-AKT-MPs | 3.0 | 3.3 | 2.7 | 0 | 28.0 |
| 3Co-AKT-MPs | 3.0 | 3.3 | 2.7 | 0 | 56.0 |
| 4Co-AKT-MPs | 3.0 | 3.3 | 2.7 | 0 | 112.0 |

**Table S2. Primer sequences of real-time PCR analysis**

| **Gene name** | **Forward primer** | **Reverse primer** |
| --- | --- | --- |
| TNF-α | CGTCAGCCGATTTGCTATCT | CGGACTCCGCAAAGTCTAAG |
| IL-1β | ACTCATTGTGGCTGTGGAGA | AGCCTGTAGTGCAGTTGTCT |
| IL-6 | AGTTGCCTTCTTGGGACTGA | TCCACGATTTCCCAGAGAAC |
| MMP13 | TGACCTCCACAGTTGACAGG | ATCAGGCACTCCACATCTTGG |
| COL-2a | CAAGAACAGCAACGAGTACCG | GTCACTGGTCAACTCCAGCAC |
| β-actin | TCCTCCTGAGCGCAAGTACTCT | CGGACTCATCGTACTCCTGCTT |
